# Supplementary material for: Making experimental data tables in the life sciences more FAIR: a pragmatic approach
Source: Gigascience. 2020 Dec 15;9(12):giaa144. doi: 10.1093/gigascience/giaa144 (PMC7736789; doi:10.1093/gigascience/giaa144)
Supplement: giaa144_GIGA-D-20-00198_Original_Submission [file giaa144_giga-d-20-00198_original_submission.pdf]

GigaScience

Towards FAIRification of experimental data tables in life sciences: a pragmatic approach

--Manuscript Draft--

**Manuscript Number:**	GIGA-D-20-00198
**Full Title:**	Towards FAIRification of experimental data tables in life sciences: a pragmatic approach
**Article Type:**	Commentary
**Funding Information:**	Agence Nationale de la Recherche (ANR-09-SYSB-003) — Dr Yves Gibon
**Abstract:**	Making data compliant with the FAIR Data principles (Findable, Accessible, Interoperable, Reusable) is still a challenge for many researchers, who are not sure which criteria should be met first and how. Illustrated from experimental data tables associated with a Design of Experiments, we propose an approach that can serve as a model for a research data management that allows researchers to disseminate their data by satisfying the main FAIR criteria without insurmountable efforts. More importantly, this approach aims to facilitate the FAIRification process by providing researchers with tools to improve their data management practices.
**Corresponding Author:**	Daniel Jacob<br>INRAE Centre de Recherche de Bordeaux-Aquitaine<br>Villenave d'Ornon, FRANCE
**Corresponding Author Secondary Information:**	
**Corresponding Author's Institution:**	INRAE Centre de Recherche de Bordeaux-Aquitaine
**Corresponding Author's Secondary Institution:**	
**First Author:**	Daniel Jacob
**First Author Secondary Information:**	
**Order of Authors:**	Daniel Jacob
	Romain David
	Sophie Aubin
	Yves Gibon
**Order of Authors Secondary Information:**	
**Additional Information:**	
**Question**	**Response**
Are you submitting this manuscript to a special series or article collection?	No
**Experimental design and statistics**<br><br>Full details of the experimental design and statistical methods used should be given in the Methods section, as detailed in our Minimum Standards Reporting Checklist. Information essential to interpreting the data presented should be made available in the figure legends.	No

Powered by Editorial Manager® and ProduXion Manager® from Aries Systems Corporation

Have you included all the information requested in your manuscript?	
If not, please give reasons for any omissions below.<br><br>as follow-up to "**Experimental design and statistics**<br><br>Full details of the experimental design and statistical methods used should be given in the Methods section, as detailed in our Minimum Standards Reporting Checklist. Information essential to interpreting the data presented should be made available in the figure legends.<br><br>Have you included all the information requested in your manuscript?<br><br>"	The data mentioned in this paper have already been published in several articles. This paper does not concern statistical processing about data but mainly their management.
**Resources**<br><br>A description of all resources used, including antibodies, cell lines, animals and software tools, with enough information to allow them to be uniquely identified, should be included in the Methods section. Authors are strongly encouraged to cite Research Resource Identifiers (RRIDs) for antibodies, model organisms and tools, where possible.<br><br>Have you included the information requested as detailed in our Minimum Standards Reporting Checklist?	Yes
**Availability of data and materials**<br><br>All datasets and code on which the conclusions of the paper rely must be either included in your submission or deposited in publicly available repositories	Yes

(where available and ethically appropriate), referencing such data using a unique identifier in the references and in the “Availability of Data and Materials” section of your manuscript.<br><br>Have you have met the above requirement as detailed in our Minimum Standards Reporting Checklist?	

Towards FAIRification of experimental data tables in life sciences: a pragmatic approach

Daniel Jacob[1,2*], Romain David[3,4], Sophie Aubin[5] , Yves Gibon[1,2]

[*]Corresponding author

Institutional addresses:

[1] INRAE, Université de Bordeaux, UMR BFP, 71 av E Bourlaux, 33140 Villenave d'Ornon, France

[2] PMB-Metabolome, INRAE, 2018. Bordeaux Metabolome Facility, MetaboHUB, 33140 Villenave d'Ornon, France. doi: 10.15454/1.5572412770331912E12

[3] INRAE, Montpellier SupAgro, Université de Montpellier, UMR MISTEA, 2, place Pierre Viala, 34060 Montpellier Cedex 2, France

[4] European Research Infrastructure on Highly Pathogenic Agents (ERINHA-AISBL), 101 rue de Tolbiac, 75013 Paris, France

[5] INRAE, DipSO, 42 rue Georges Morel, 49070 Beaucouzé, France,

Email addresses, ORCID :

DJ: daniel.jacob@inrae.fr, ORCID: 0000-0002-6687-7169

RD: romain.david@erinha.eu, ORCID: 0000-0003-4073-7456

SA: sophie.aubin@inrae.fr ORCID: 0000-0003-4805-8220

YG: yves.gibon@inrae.fr ORCID: 0000-0001-8161-1089

Abstract

Making data compliant with the FAIR Data principles (Findable, Accessible, Interoperable, Reusable) is still a challenge for many researchers, who are not sure which criteria should be met first and how. Illustrated from experimental data tables associated with a Design of Experiments, we propose an approach that can serve as a model for a research data management that allows researchers to disseminate their data by satisfying the main FAIR criteria without insurmountable efforts. More importantly, this approach aims to facilitate the FAIRification process by providing researchers with tools to improve their data management practices.

Keywords: Research Data Management; FAIR Data principles; FAIR assessment; experimental data tables

Background

The publication of research data according to the FAIR principles [1] has become a major challenge with the aim of integrating them into the overall research process (e.g. European commission explicitly mentions FAIR principles as a mandatory reference [2]). However, implementing these principles is not so easy and requires changes in data management practices. According to Jacobsen et al [3], the FAIR principles can be seen as a consolidation of good data management practices to extend management to the notion of machine reuse of data. Thus, it seems appropriate to use virtuous principles as far upstream as possible from the data rather than trying to comply with FAIR principles downstream. This is the starting point of the approach we propose in order to integrate these principles into the practices of researchers.

Set the scene

Let's take a concrete example from the plant biology domain. A study on the metabolism of tomato fruits [4] involved growing several hundred tomato plants in greenhouses. This multifactorial experiment (stages of development and type of treatment applied to each group of plants) generated a dozen large experimental data tables. Part of the data was acquired in the greenhouse manually using spreadsheets. While another part of the data comes from biochemical and metabolomics analyses, carried out on the thousand samples taken and returned in the form of data tables weeks or even months later.

The use of spreadsheets is therefore central here, being the tool par excellence for researchers. However, many manual handling is required to link together experimental data tables from several analytical techniques, according to samples. Such repeated data handling, a potential source of errors, can compromise the consistency of data, which must be managed throughout the study by ensuring that each analysis is well linked to its sample. In addition, this study involved several partners who wanted to access the data as soon as possible. We needed to review our data

management practices. The question remained as to how to motivate and convince researchers to change their practice a little.

To promote good practices, provide services

To bring about a change in habits, an improvement in the gains/efforts ratio can act as a motivational lever. This is precisely the basis of our approach: to provide tools and services that are truly useful to researchers in order to get them to adopt good practices. Thus, a tool allowing to automatically combine data sets according to a common attribute (identifiers) would be easily adopted because it would allow several types of variables to be analyzed according to different parameters without any tedious data handling. Now, it is very likely that the spreadsheet will continue to be used despite its drawbacks, in particular the lack of constraint on information structure that, paradoxically, makes it so popular. Rather, an approach is needed that largely avoids such drawbacks. In this perspective, we propose a data structuring similar to data dictionaries that is easy to implement by the researchers themselves, because essentially using a spreadsheet (**Figure 1**). Structural metadata (*e.g.* links between data tables) are described, together with unambiguous definitions of all internal elements (e.g. column definitions along with their semantic definition), through links to accessible definitions, such as community-approved ontologies where possible, as recommended by Jacobsen et al [3]. The change in practice consists mainly in doing this structuring work upstream of any data processing, knowing that from this structuring, the researchers can thus have tools at their disposal to avoid very time-consuming, repetitive and tedious tasks later on. Better still, it opens up data on a whole ecosystem of potential applications, according to their needs and skills (**Figure 2**).

The advantage of this approach is manifold. It allows the data to be structured in such a way that the researcher *i*) can proceed step by step as the data become available and *ii*) can easily exploit it with tools immediately afterwards. In doing so, the researcher performs a data FAIRification in

order to handle it more efficiently and not only for publishing it. Thus, it is the FAIRification of data that is integrated by design into the data processing workflow. In addition, from the structured metadata it becomes possible to convert them directly into a standard format. In our case, we used a data format based on a community, open, interoperability standard [5]. Slightly adapted to our needs *e.g.* by specifying the category of each attribute, it now enables to disseminate the data according to an open schema that greatly facilitates the reuse of the data by machines (**Figure 3**). Data generation according to this open schema is included in the proposed tools and does not require additional effort for the researcher. So, in our approach data FAIRification is closely related to data management, avoiding a retroactive process that would require more time, costs and computer skills [6,7].

Publication of data

When it comes to publishing their results, researchers usually provide the minimum required to support their claims [8]. This generally results in a loss of data (quantitative aspect) and information (qualitative aspect) compared to the totality of the data acquired during the study. However, journal editors and reviewers are increasingly recommending that all data, complete and non-synthetic, generated during the course of the study be made available. We believe that our approach should facilitate this dissemination because the work has been done upstream, and that when needed the data can be deposited in an appropriate repository quickly enough without having to do data archaeology, while at the same time meeting the essential criteria of the FAIR principles is guaranteed (**Additional files 1,2 and 3**). In addition, the expected benefits are numerous, including exploiting the full potential of the data sets, improving the reproducibility and reliability of the data, but also increasing visibility and citation due to the reuse of the data by both humans and machines.

Furthermore, a lever of motivation would be the recognition of this effort to publish data according to the FAIR principles. Unfortunately, researchers who devote time and expertise to activities like data curation are not currently rewarded by traditional career progression metrics. We believe that this should change in the future, and crediting and rewarding mechanisms are the subject of the Research Data Alliance SHAring Rewards and Credit Interest Group [9].

Summary of the proposed approach and beyond

1. To provide researchers with services that are truly useful, time-saving and efficient. Care must also be taken not to deprive them of their know-how or trap them in turnkey solutions that prevent any opportunity of testing several hypotheses or scenarios. Rather, we need to open the data to a whole ecosystem of software possibilities.
2. They are the ones who have the best control and understanding over their data, so they are in the best position to annotate it. On the other hand, it is necessary to assist them as much as possible in this process, by proposing protocols and methods adapted to their computer skills, a domain which is not their core expertise. In particular, dictionaries of vocabulary corresponding to their field and frequently used should be provided in order to standardize the annotations as much as possible.
3. Behind a dataset, there is often an involved team with a wide range of skill levels. We must take into account their way of working, their work habits; so instead of wanting to change their habits completely, we must rather adapt them in a way that is beneficial to them, and make them actors in the data FAIRification process. Probably the best FAIR training is the one based on their data. But it is necessary to capitalize on good practices in written protocols, and referenced into data management plans.
4. Researchers must be involved in the data FAIRification process by providing them with tools for assessing their practices so that they can progressively improve them in stages,

having properly integrated each of the criteria implemented. Especially, these assessment tools should highlight all the steps where small actions can significantly improve the FAIRification of the data. They will thus be more inclined to integrate them into their practice with full knowledge of the facts.

5. Finally, a last word on the data provenance: not only the authors, but above all the context, the methods of data acquisition and processing are crucial information for a good reuse. Unfortunately, this aspect is somewhat neglected, if not absent. An effort still needs to be made in this direction in order to sensitize and motivate data producers.

Declarations

Ethics approval and consent to participate

Not applicable.

Consent for publication

Not applicable.

Availability of data and material

All information, documents, data and software concerning ODAM are accessible from Github [10]

Competing interests

The authors declare that they have no competing interests

Funding

DJ, RD and YG were partly supported by the PHENOME-EMPHASIS project funded by the French National Research Agency (ANR-11-INBS-0012). DJ and YG were also supported by the FRIMOUSS project funded by the French National Research Agency (ANR-15-CE20-0009-01). RD was also partly supported by the EPPN2020 project (H2020 grant N°731013), the EOSC-Life european program (grant agreement N°824087). SA was partly supported by the FooSIN project funded by the French National Research Agency (ANR□19□DATA□0019-01). All authors were

also partly funded by the French National Research Institute for Agriculture, Food and the Environment (INRAE). The FRIM1 dataset came from research supported by the Eranet Erasysbio+ FRIM project funded by the French National Research Agency (ANR-09-SYSB-003) and the MetaboHUB project funded by the French National Research Agency (ANR-11-INBS-0010).

Author contributions

conceptualization: D.J; data curation: D.J; funding acquisition: Y.G; methodology: D.J, R.D; software: D.J; writing—original draft: D.J, R.D; writing—review and editing: D.J, R.D, S.A, Y.G. All authors read and approved the final manuscript.

Acknowledgements

We thank Catherine Deborde (PMB-Metabolome, INRAE, MetaboHUB) for advice on the manuscript and for constructive reviews.

References

1. Wilkinson MD, Dumontier M, Aalbersberg IJJ, Appleton G, Axton M, Baak A, et al. The FAIR Guiding Principles for scientific data management and stewardship. Sci Data. 2016;3:160018. doi:10.1038/sdata.2016.18.
2. European Commission Directorate General for Research and Innovation (2018) Turning FAIR into reality, Final Report and Action Plan from the European Commission Expert Group on FAIR Data, https://ec.europa.eu/info/publications/turning-fair-reality_en [Accessed June 04, 2020]
3. Jacobsen A, de Miranda Azevedo R, Juty N, Batista D, Coles S, Cornet R et al (2020) Data Intelligence 2: 1-2, 10-29 doi:10.1162/dint_r_00024
4. Bénard C, Biais B, Ballias P, Beauvoi B, Bernillon S, Cabasson C et al (2018), FRIM - Fruit Integrative Modelling, doi.org/10.15454/95JUTK, Portail Data INRAE, V3

5. Data Package, Frictionless Data Specs https://specs.frictionlessdata.io/data-package/ [Accessed June 04, 2020]
6. Rocca-Serra P and Sansone SA (2019) Experiment design driven FAIRification of omics data matrices, an exemplar, Scientific Data volume 6, 271 doi:10.1038/s41597-019-0286-0
7. European Commission, Directorate-General for Research and Innovation (2018), Cost-Benefit analysis for FAIR research data - Cost of not having FAIR research data, https://op.europa.eu/en/publication-detail/-/publication/d375368c-1a0a-11e9-8d04-01aa75ed71a1 [Accessed June 04, 2020]
8. Leonelli S, Smirnoff N, Moor J (2013) Making open data work for plant scientists, Journal of Experimental Botany, pp. 4109–4117, doi:10.1093/jxb/ert273
9. David R, Mabile L, Specht A, Stryeck S, Thomsen M, Yahia M et al. (2020) FAIRness Literacy: the Achilles' Heel of applying FAIR Principles. Hal-02483307 https://hal.inrae.fr/hal-02483307
10. "ODAM: Deployment and User's Guide". GitHub 2020 https://inrae.github.io/ODAM/

Figures

Figure 1: ODAM-compliant structural metadata associated with the FRIM experimental data files: (**A**) Since all the experimental data tables were generated as part of an experiment associated with a Design of Experiment (DoE), each file thus contains data acquired sequentially as the experiment progressed. There must therefore be a link between each file, i.e. information that connects them together. In most cases, this information corresponds to identifiers. Thus, we can depict all data tables as a tree, the implicit link that connects them together being "obtained from''. We need two specific files (**B &C**) to describe the structural metadata of the whole dataset, where only a part of each of them are shown here. (**B**) This metadata file makes it possible to associate each data subset with a key concept corresponding to the main entity of the subset, each subset being stored as a file (the grey rectangle). It also defines for each subset the link to the subset it originates (grey arrows). (**C**) This metadata file allows to annotate each

attribute (concept/variable) with some minimal but relevant metadata, such as: its description with its unit, the data type, but also its category. (**D**) The category defined by controlled vocabulary (CV) is used to specify the type of each variable. In each of these two files (entities and attributes), it is possible to annotate each of the terms with unambiguous definitions through links to accessible (standard) definitions. Thus constructed, this metadata constitutes a dictionary describing each file (entity) as well as all the columns of the tables (attributes) offering a better guarantee in the correct (re)use of the data for users who have not produced these data. See Data Preparation Protocol for ODAM Compliance for more details (doi:10.17504/protocols.io.betcjeiw).

Figure 2: ODAM software suite: In light blue (promote <-> provide) the engine of the approach, in purple the data and metadata provided by the user, in dark blue the activities related to the life cycle of the data. The whole process is implemented primarily to make better use of its data before its dissemination. The ODAM software embeds an API (Application Programming Interface) layer that allows interoperability between the different tables and the applications that will be able to use them. With the help of this layer, it opens up a whole ecosystem of potential applications, depending on your needs but also on your skills in the proposed tools. From the set of data files (which are non-combined tables, each corresponding to a particular observational unit that we name an entity), the user can: 1) Visualize the data associated with their metadata according to several criteria and in a completely interactive way with the help of the data explorer. 2) Export in tabular form subsets selected according to his criteria with combined, merged data. 3) Build and test his models more easily using a scripting language such as R, which allows it to repeat different scenarios according to a variety of parameters. An R package allows to perform extractions according to the same criteria as those proposed in the data explorer. The benefit of this approach is that the "life of the data" is integrated into the scientific research process, according to good data management practices that meet the essential FAIR criteria. The FAIRification can be considered from two points of

view: 1/ It is linked to the data life cycle by the annotations and curations made on the data themselves, and to the quantity and quality of the information associated with the data (protocols, publications, keywords, ...), 2/ it can also be considered from the point of view of its data management practices, which must improve over time, which is precisely what the FAIR assessment grids attempt to measure, and more particularly the reproducibility and reusability of the data. See ODAM Deployment and User's Guide for more details (https://inrae.github.io/ODAM/).

Figure 3: Interconnection of the different elements of the FRIM dataset from the Data INRAE repository as a hub (based on Dataverse https://dataverse.org/). By relying on explicit schemas (JSON-LD, JSON Schema) for both metadata and data, it becomes possible to reuse the data without friction, both by humans and machines. The use of spreadsheets greatly facilitates the annotation of data with metadata by the data producers themselves. Thus, this is technology, however powerful, that becomes part of the practices of non-experts in the domain, not the other way around. In addition, this further enhances the FAIR criteria, especially the reuse and interoperability criteria. We used three FAIR grids, very different from each other. The first one, the OZONOME 5-star0   data ranking tool, aims to perform an evaluation based on the FAIR principles as defined by Willkinson et al. (2016). The main result is an overall rating, indicating the overall fairness of the data set. The other two grids are dedicated to a more refined assessment. The Fair Data Maturity Model (FDMM) document describes a maturity model for the FAIR assessment with indicators, priorities and assessment methods, which are useful for standardizing assessment approaches in order to allow comparison of their results. Whereas the FAIR SHARC (SHAring Rewards and Credit) document allows the fairness of projects and associated human processes to be assessed, either by external evaluators or by the researchers themselves. Therefore, these grids cannot be compared with each other, but rather complement each other. Overall, the FAIRness of our dataset using the ODAM+Dataverse combination is of a good standard. However, to achieve complete

FAIRification, we need to move towards semantic web approaches. By relying on explicit data schemas, the effort to climb this mountain can be envisaged with less fear.

Additional files

Additional file 1. FAIR evaluation of the FRIM1 dataset according to the 5 ★ Data Rating Tool grid. It aims to perform an evaluation based on the FAIR principles as defined by Willkinson et al. [1]. The main result is an overall rating, indicating the overall fairness of the data set.

Additional file 2. FAIR assessment of the FRIM1 dataset according to the FDMM (FAIR Data Maturity Model) grid. This document describes a maturity model for the FAIR assessment with indicators, priorities and assessment methods, which are useful for standardizing assessment approaches in order to allow comparison of their results.

Additional file 3. FAIR assessment of the FRIM1 dataset according to the SHARC (Sharing Rewards and Credit) grid. This document allows the fairness of projects and associated human processes to be assessed, either by external evaluators or by the researchers themselves.

Figure 1:

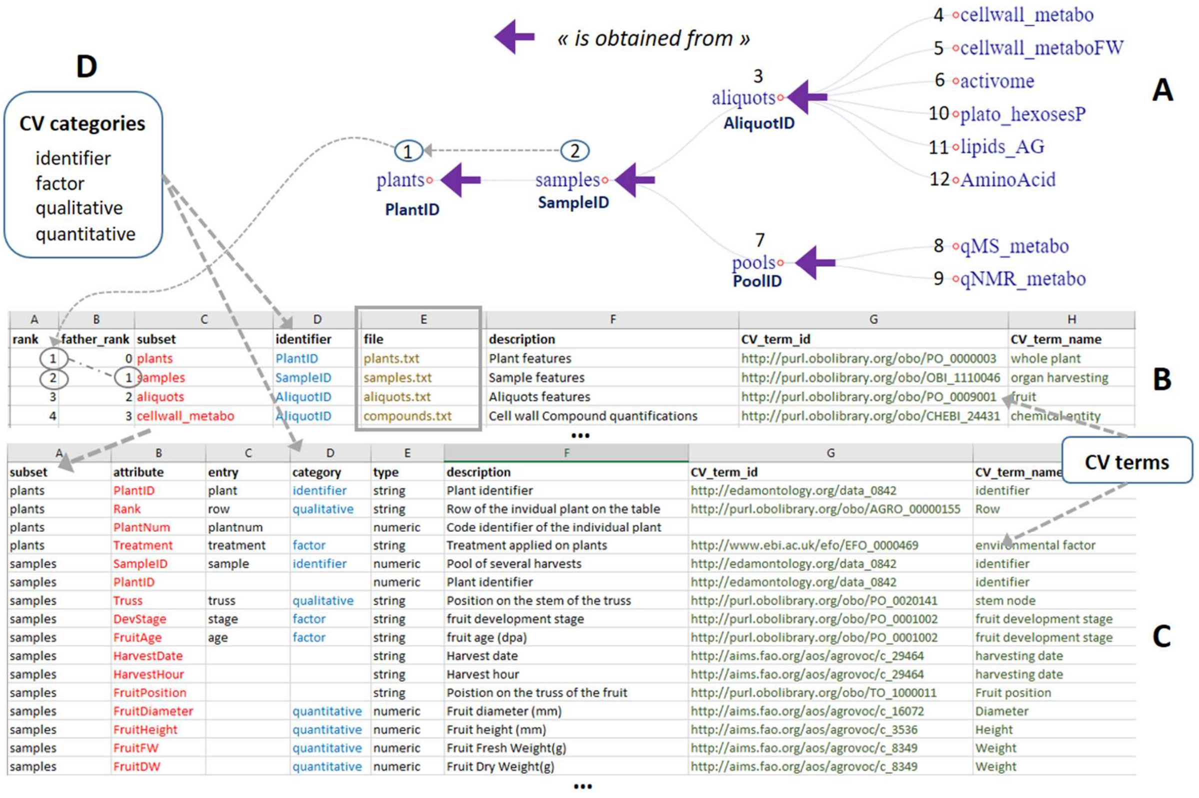

Figure 2:

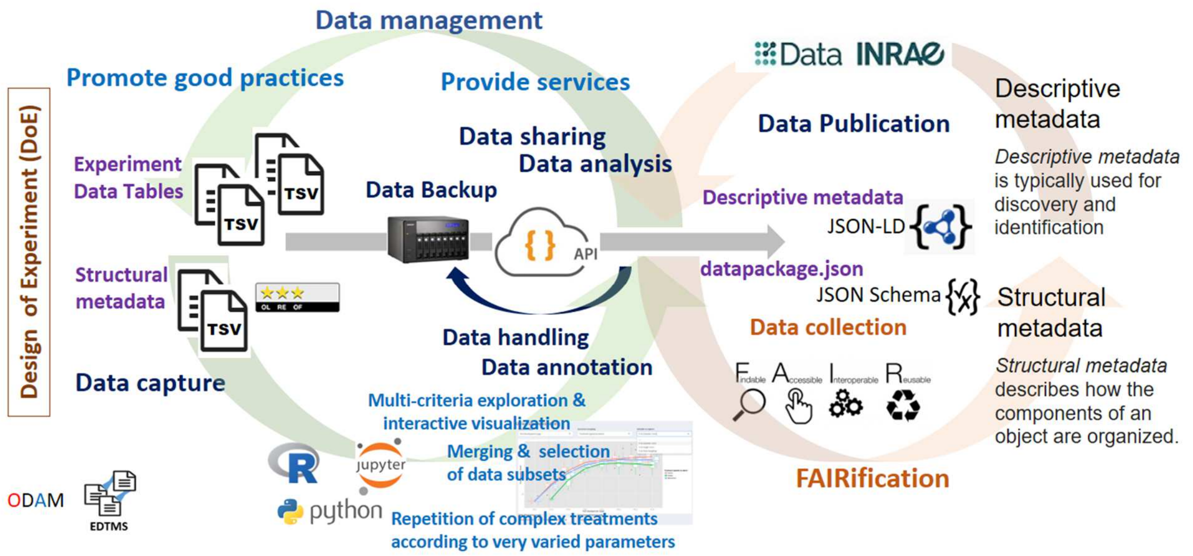

Figure 3:

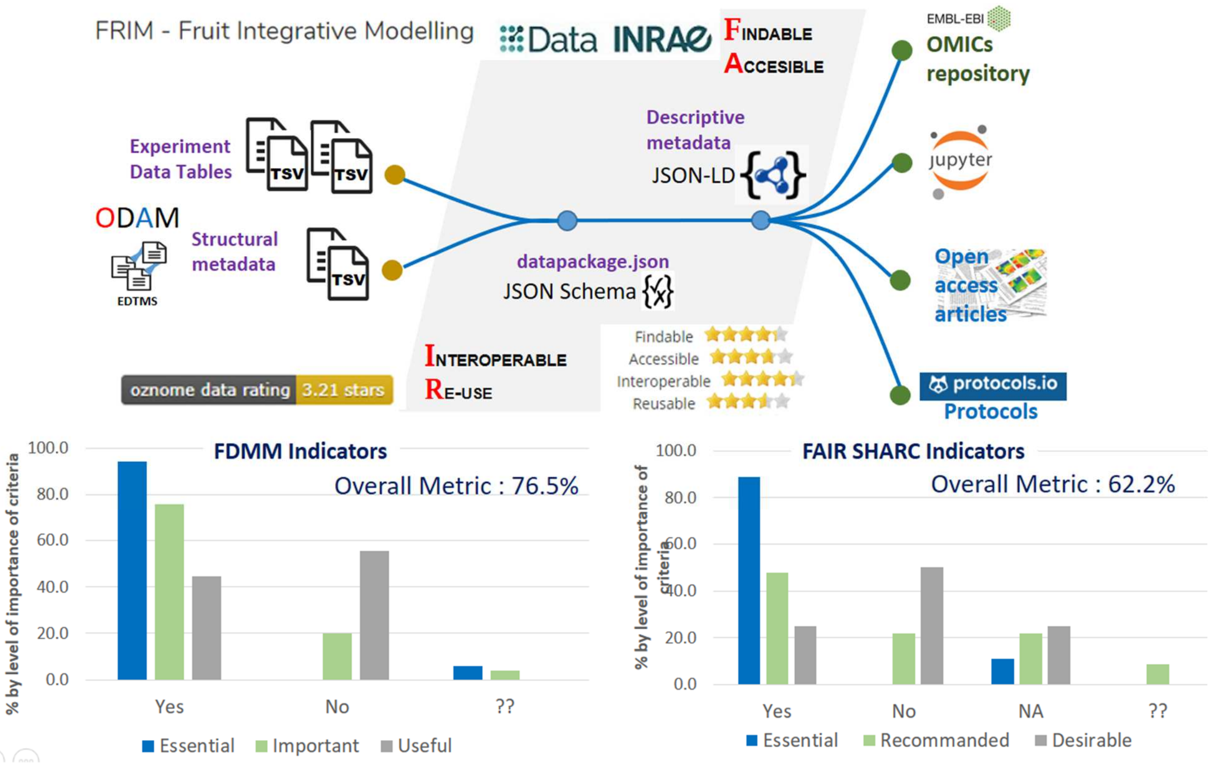
FRIM - Fruit Integrative Modelling
Data INRAE
FINDABLE
ACCESIBLE
EMBL-EBI
OMICs repository
Experiment Data Tables
TSV
Descriptive metadata
JSON-LD
jupyter
ODAM
Structural metadata
EDTMS
datapackage.json
JSON Schema
Open access articles
Findable
Accessible
Interoperable
Reusable
INTEROPERABLE
RE-USE
oznome data rating 3.21 stars
protocols.io
Protocols
FDMM Indicators
Overall Metric : 76.5%
% by level of importance of criteria
Yes
No
??
Essential
Important
Useful
FAIR SHARC Indicators
Overall Metric : 62.2%
% by level of importance of criteria
Yes
No
NA
??
Essential
Recommanded
Desirable

280

281

Figure 1

Click here to access/download;Figure;Figure1.png

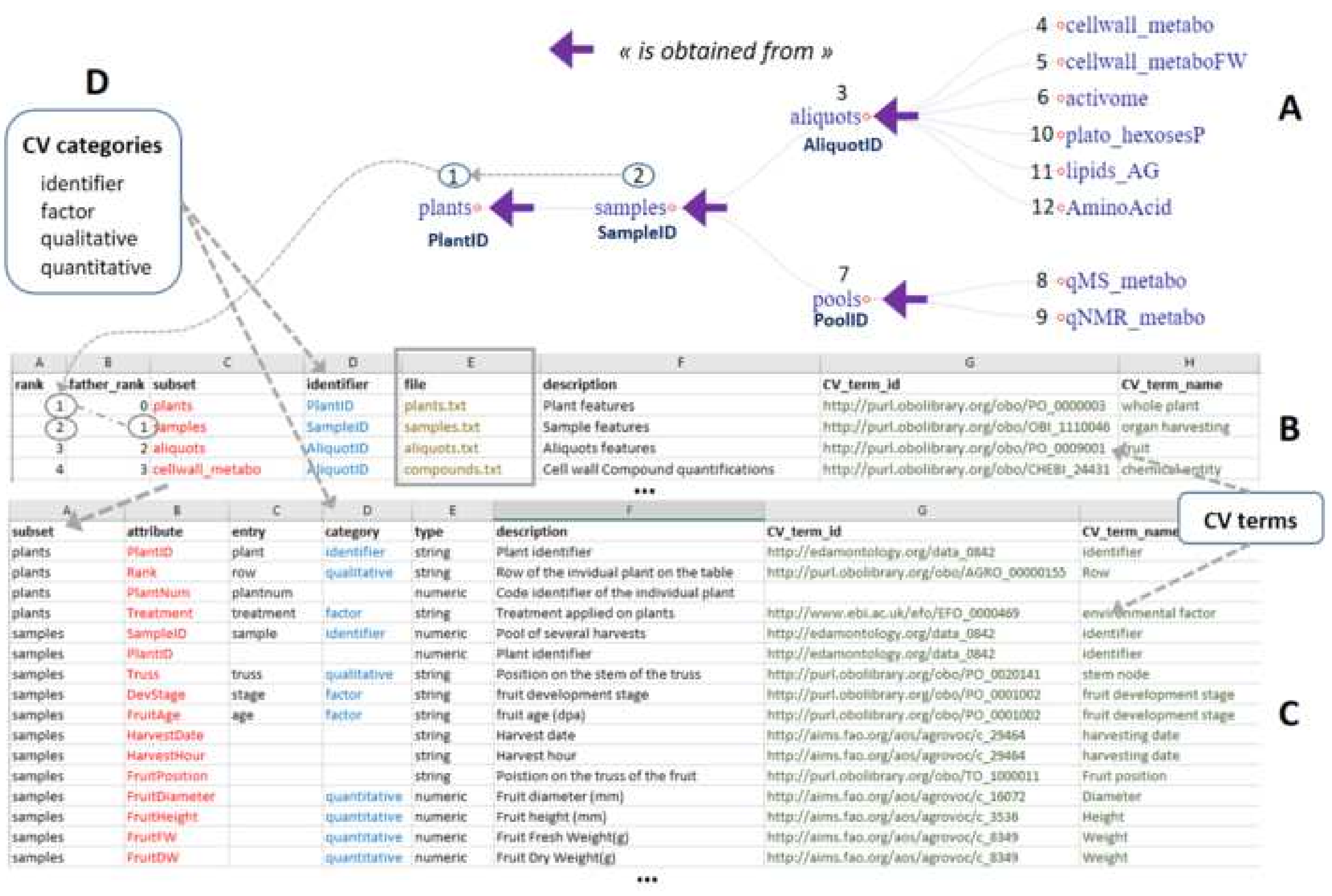

A: rank	B: father_rank	C: subset	D: identifier	E: file	F: description	G: CV_term_id	H: CV_term_name
1	0	plants	PlantID	plants.txt	Plant features	http://purl.obolibrary.org/obo/PO_0000003	whole plant
2	1	samples	SampleID	samples.txt	Sample features	http://purl.obolibrary.org/obo/OBI_1110046	organ harvesting
3	2	aliquots	AliquotID	aliquots.txt	Aliquots features	http://purl.obolibrary.org/obo/PO_0009001	fruit
4	3	cellwall_metabo	AliquotID	compounds.txt	Cell wall Compound quantifications	http://purl.obolibrary.org/obo/CHEBI_24431	chemical entity

...

A: subset	B: attribute	C: entry	D: category	E: type	F: description	G: CV_term_id	CV_term_name
plants	PlantID	plant	identifier	string	Plant identifier	http://edamontology.org/data_0842	identifier
plants	Rank	row	qualitative	string	Row of the invidual plant on the table	http://purl.obolibrary.org/obo/AGRO_00000155	Row
plants	PlantNum	plantnum		numeric	Code identifier of the individual plant		
plants	Treatment	treatment	factor	string	Treatment applied on plants	http://www.ebi.ac.uk/efo/EFO_0000469	environmental factor
samples	SampleID	sample	identifier	numeric	Pool of several harvests	http://edamontology.org/data_0842	identifier
samples	PlantID			numeric	Plant identifier	http://edamontology.org/data_0842	identifier
samples	Truss	truss	qualitative	string	Position on the stem of the truss	http://purl.obolibrary.org/obo/PO_0020141	stem node
samples	DevStage	stage	factor	string	fruit development stage	http://purl.obolibrary.org/obo/PO_0001002	fruit development stage
samples	FruitAge	age	factor	string	fruit age (dpa)	http://purl.obolibrary.org/obo/PO_0001002	fruit development stage
samples	HarvestDate			string	Harvest date	http://aims.fao.org/aos/agrovoc/c_29464	harvesting date
samples	HarvestHour			string	Harvest hour	http://aims.fao.org/aos/agrovoc/c_29464	harvesting date
samples	FruitPosition			string	Poistion on the truss of the fruit	http://purl.obolibrary.org/obo/TO_1000011	Fruit position
samples	FruitDiameter		quantitative	numeric	Fruit diameter (mm)	http://aims.fao.org/aos/agrovoc/c_16072	Diameter
samples	FruitHeight		quantitative	numeric	Fruit height (mm)	http://aims.fao.org/aos/agrovoc/c_3536	Height
samples	FruitFW		quantitative	numeric	Fruit Fresh Weight(g)	http://aims.fao.org/aos/agrovoc/c_8349	Weight
samples	FruitDW		quantitative	numeric	Fruit Dry Weight(g)	http://aims.fao.org/aos/agrovoc/c_8349	Weight

...

Figure 2

Click here to access/download;Figure;Figure2.png

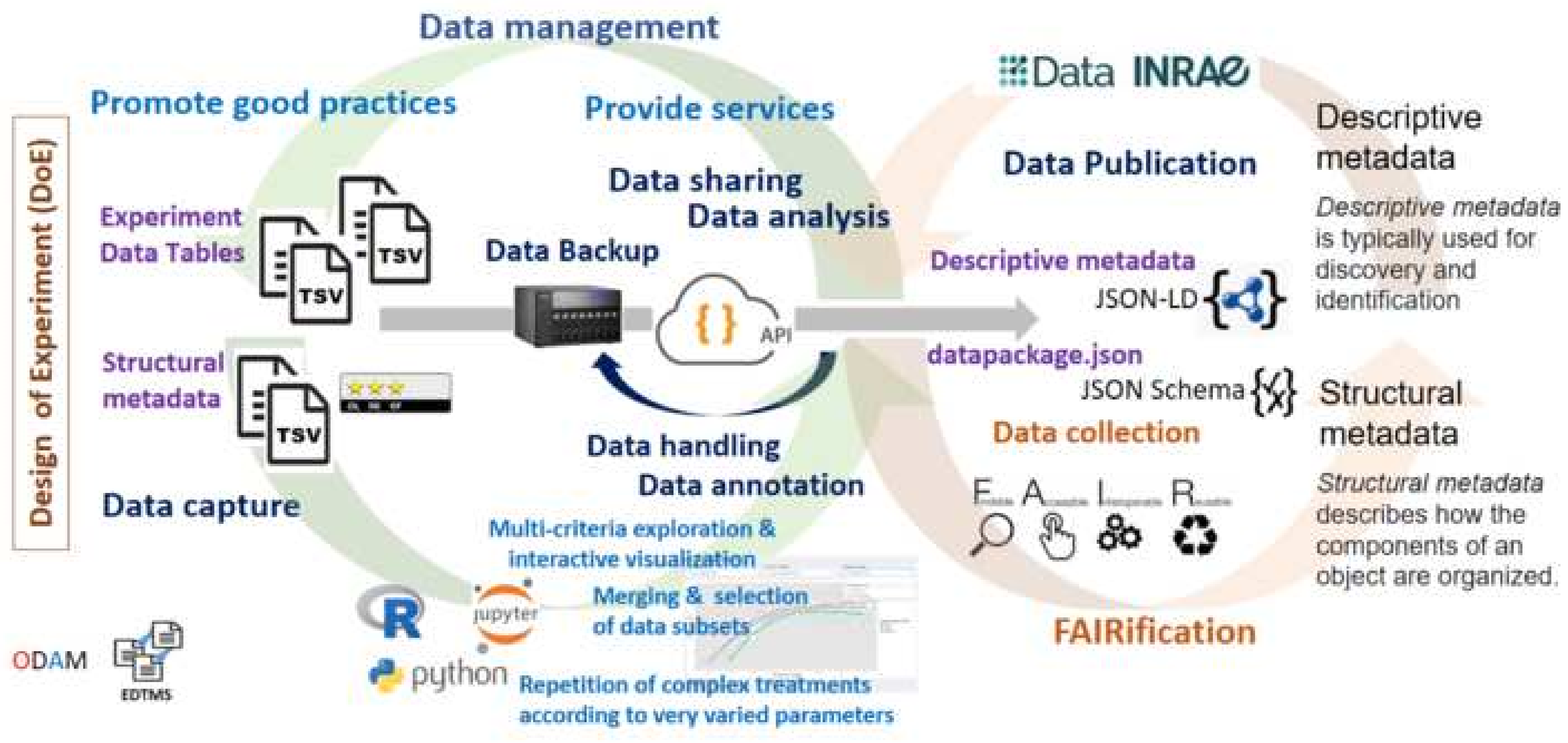

Figure 3 Click here to access/download;Figure;Figure3.png

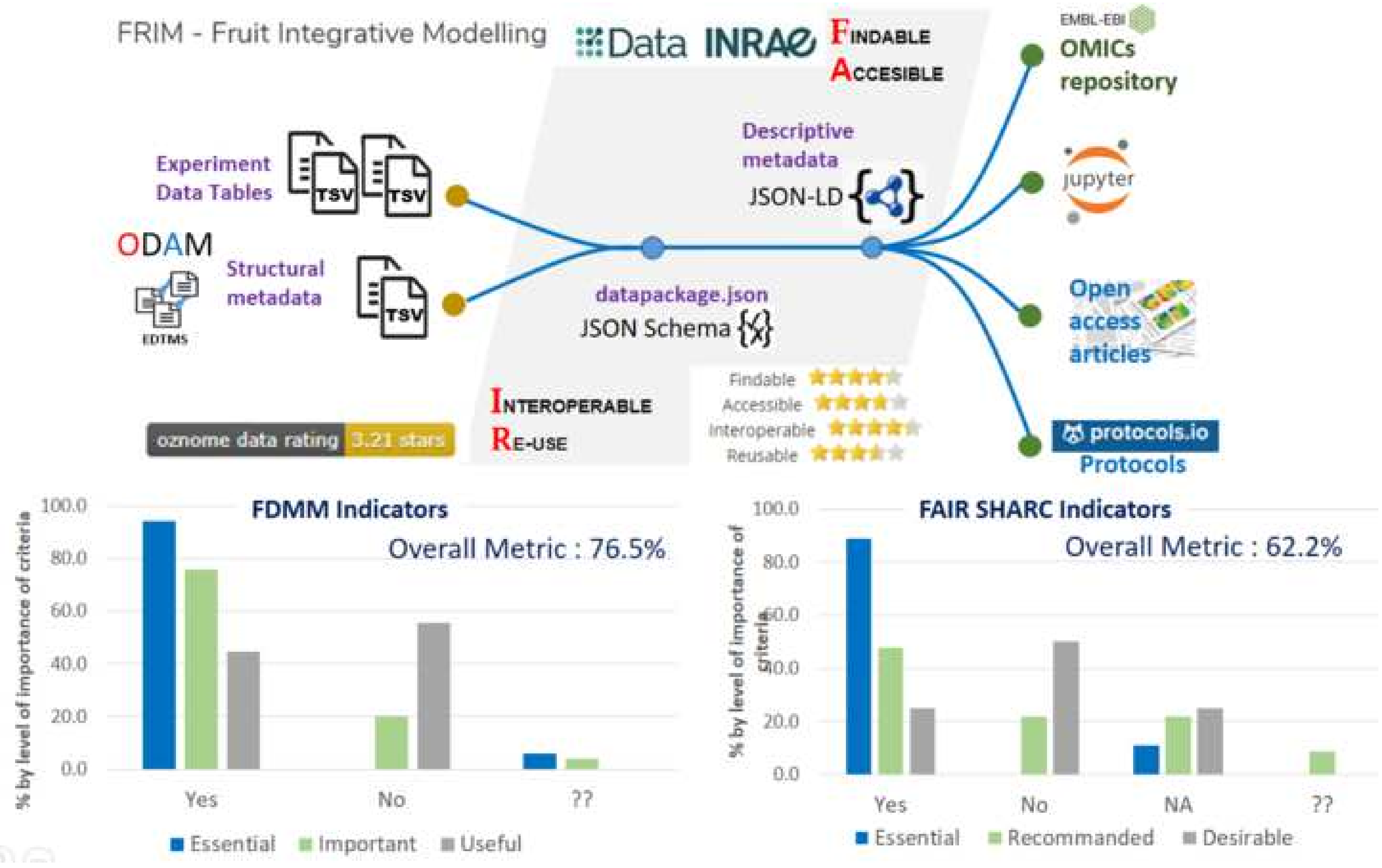

Additionnal file 1

Click here to access/download

Supplementary Material

Additional_file_01.xlsx

Additionnal file 2

Click here to access/download
Supplementary Material
Additional_file_02.xlsx

Additionnal file 3

Click here to access/download
Supplementary Material
Additional_file_03.xlsx
