# Supplementary material for: Making experimental data tables in the life sciences more FAIR: a pragmatic approach
Source: Gigascience. 2020 Dec 15;9(12):giaa144. doi: 10.1093/gigascience/giaa144 (PMC7736789; doi:10.1093/gigascience/giaa144)
Supplement: giaa144_GIGA-D-20-00198_Revision_1 [file giaa144_giga-d-20-00198_revision_1.pdf]

GigaScience

Towards FAIRification of experimental data tables in life sciences: a pragmatic approach

--Manuscript Draft--

**Manuscript Number:**	GIGA-D-20-00198R1
**Full Title:**	Towards FAIRification of experimental data tables in life sciences: a pragmatic approach
**Article Type:**	Commentary
**Funding Information:**	Agence Nationale de la Recherche (ANR-09-SYSB-003) — Dr Yves Gibon
**Abstract:**	Making data compliant with the FAIR Data principles (Findable, Accessible, Interoperable, Reusable) is still a challenge for many researchers, who are not sure which criteria should be met first and how. Illustrated from experimental data tables associated with a Design of Experiments, we propose an approach that can serve as a model for a research data management that allows researchers to disseminate their data by satisfying the main FAIR criteria without insurmountable efforts. More importantly, this approach aims to facilitate the FAIRification process by providing researchers with tools to improve their data management practices.
**Corresponding Author:**	Daniel Jacob<br>INRAE Centre de Recherche de Bordeaux-Aquitaine<br>Villenave d'Ornon, FRANCE
**Corresponding Author Secondary Information:**	
**Corresponding Author's Institution:**	INRAE Centre de Recherche de Bordeaux-Aquitaine
**Corresponding Author's Secondary Institution:**	
**First Author:**	Daniel Jacob
**First Author Secondary Information:**	
**Order of Authors:**	Daniel Jacob
	Romain David
	Sophie Aubin
	Yves Gibon
**Order of Authors Secondary Information:**	
**Response to Reviewers:**	Dear Editor,<br><br>Concerning manuscript "Towards FAIRification of experimental data tables in life sciences: a pragmatic approach" (GIGA-D-20-00198), all remarks of reviewers 1 and 3 have been taken into account. A response was also made to reviewer 2 for whom we think his judgment seems a bit hasty but we ve taken in account his remarks as much as possible by rewording.<br>Reviewers 1 and 3, experts in the field, provided us with valuable revision elements to improve the manuscript. We were keen to satisfy the various advice and remarks, especially since they were provided in a spirit of benevolence with a real interest for the content of the manuscript. The type of paper being a commentary, i.e. a short paper, we wanted to keep the initial structure of the manuscript, i.e. a more general argument in the main text, and more technical details in the figures and their captions. Thus, considering the limit imposed (1500 words, 10 references), we nevertheless tried to answer as much as possible to the whole advices and suggestions.<br>The answers and changes that have been made are detailed below.<br><br>Please let us know if some additional modifications are needed.

Powered by Editorial Manager® and ProduXion Manager® from Aries Systems Corporation

Best wishes,

Daniel Jacob

Reviewer reports:
=============

Reviewer #1:

Dear Authors,

I enjoyed your manuscript very much, as it addresses the difficult realities of trying to bring FAIRness to the source of the data! As the institutional data steward for my institute, I resonated very much with the issues you discussed in this submission.

Answer: We are very pleased with your interest in our work.

I have a few comments that might improve the submission in several ways.

1) I don't think you have given sufficient acknowledgement of prior art, especially around the integration of semantic data capture into spreadsheets. Acknowledging (at least) the Right Field project and the ISA-toolkit would be appropriate (and maybe even discussing if Right Field/ISA-Tools can work in-parallel with the structured approach you refer-to in the protocols.io submission, Figure 1 legend)

Answer: Indeed, although being an important point we neglected it a bit in the first version. Partly because this point is still under development regarding our approach (cf https://inrae.github.io/ODAM/todo/). We have mentioned this point at the end of the Figure 1 legend by citing the suggested tools plus another very promising one as well.

2) Regarding the protocols.io submission that you refer-to in the Figure 1 legend - this is a very significant contribution, in my opinion! I feel it is sufficiently important that it should probably be discussed more directly, and in more detail, in this article. I finished reading the article not fully understanding what you meant, but after reading your Data Preparation Protocol for ODAM Compliance submission to protocols.io, I had a much better understanding (and I might try to duplicate that approach in my own institute!). Unfortunately, I don't think that Figure 1 adequately explains what you are doing/proposing, and so it was a bit disappointing that I had to go to that reference to fully understand your paper.

Answer: It is true that without the associated protocol file, it was difficult to fully understand the process. Therefore, we have redone Figure 1 and completely rewritten its legend, in particular by taking up again important points mentioned in the protocol. Moreover, we have partially rewritten the " To promote good practices, provide services " section to better clarify the purpose of our work.
In addition, we have associated the protocol file as an additional file to the article so that it is now an integral part. In this way, we hope to have made our approach more readable.

3) Another piece of software that I think would be appropriate to mention is the SEEK data management platform, from the FAIR-DOM project. Project-level and dataset-level metadata and provenance capture is handled by this open-source tool quite nicely!

Answer: The strength of our approach according to us, being the structural metadata associated with the data, we wanted to focus our paper more on this point. Thus we distinguish between descriptive metadata, i.e. the overall context on the one hand and structural metadata, i.e. metadata describing the interconnections between the data along with the functional categories on the other hand. The data and their metadata formatted according to our approach allows users to have a great flexibility in the choice of data repository. We have given some examples of this in Figure 2. Regarding

the SEEK data management platform, we actually found it very relevant indeed, so we have mentioned it in the main text.

4) Numerous grammatical errors throughout. I captured some of them, but there are others that i didn't highlight:

line 29 remove "a” from "a research"
Answer: Ok, corrected.

line 57: remove "many"
Answer: Ok, corrected

line 68/69: pronoun missing "allowing to”:
Answer: As the text has changed, this is no longer applicable. But indeed, we have paid attention to the use of these verbs in English (allow X to, enable X to) in this paper. Thank you.

line 75/76: bad grammar: because essentially using a spreadsheet
Answer: Ok, corrected

line 82/83: I don't understand this sentence: Better still, it opens up data on a whole ecosystem of potential applications, according to their needs and skills
Answer: We have rewritten this sentence as this: “Besides, depending on the needs and skills of researchers, data can be used in a wide variety of ways”. See lines 87-88

lines 86-88: the sentences that span those lines needs to be grammatically corrected.
Answer: We have rewritten this sentence as this: “In doing so, FAIRification of data is carried out in order to handle data more efficiently and not just to publish it”. See lines 91-92

line 91: pronoun missing "enables to"
Answer: Corrected

line 122: the second item in this list is referring (I guess?) to "researchers", the first item in the list. This is not how the list is introduced (Summary of the proposed approach and beyond). that list item needs to be restructured to match the form of the other list items.
Answer: Ok we have rewritten this sentence. See lines 130-135

Thank you for your efforts in writing this manuscript! I wish you luck on publication!
Answer: Thank you for your encouragement, very appreciated!

Sincerely,

Mark Wilkinson

Reviewer #2:

The authors provide a short summary of ways to make data publication compliant with FAIR principles (findable, accessible, interoperable, reusable). These principles are universally desirable and intuitive -- they describe requirements for sound research and communication of findings.

The paper describes ways of fixing the problem of spreadsheets without necessarily taking the spreadsheets away from the researchers. I would not agree that they are a "tool par excellence". They are convenient -- available and with a low bar for entry. Ironically, it is this very convenience that makes them unreliable for storage and analysis.

The paper hinges on 5 points, listed in the "Summary of proposed approach". My

interpretation of these points is the following

1. Make data available through a service that is tailored to the needs of the researcher and their expertise in data wrangling.
2. Create a database schema, normalize the data and map it onto fixed vocabulary or ontology.
3. Train researchers to adopt FAIR principles a la carte and where it is both practical and useful for them to do so. Thus, as opposed to transitioning to a fully "FAIRized" system, stagger the adoption and training process and use the researcher's own data for training. Essentially, use a bit of social engineering to get people to do what you feel is good for them.
4. Have a way to assess whether implementing new data handling methods is adding benefit.
5. Every aspect of data collection, handling, analysis and publication should be designed with FAIR principles in mind.

These 5 points are true but, in my mind, they are so true that they are essentially widely accepted as necessary. For example, a fixed vocabulary and normalized database schema for data storage and lookup are common solutions to the problem of consistency and querying. I don't think that points like this need to be emphasized anymore.

The workflow shown in Figure 2 is flexible and sophisticated but illustrates steps that are required in any research venture that is sound (e.g. data backup, metadata, visualization).

Answer:

We agree overall with your analysis, except that:
The FAIR principles are widely accepted, but this does not mean that they are easily adopted by all for data dissemination (e.g. in re3data.org more than 50% of the repositories do not have a Persistent Identifier!). There is still a long way to go (way of the cross?) before we get there.
Beyond the principles, it is above all the practical aspects that make it possible to get there. It is on these aspects that our paper focuses, especially for experimental data tables. For this type of data in particular, interoperability criteria require structural metadata for fostering reuse. However, regardless of the skills required to build a data model, it seems unreasonable to us to have to build such models in order to constitute a database each time a greenhouse or field experiment is carried out. This approach is simply out of touch with the realities in most cases. This is why, in such cases, we need to be very pragmatic in proposing approaches that are easy to implement within a reasonable timeframe, and that do not necessarily require strong IT engineering skills. This is what we try to propose in this paper concerning more specifically experimental data tables.

We agree with you that "a tool par excellence" was a bit excessive. So we changed that to "a tool that researchers master very well".

Reviewer #3:

This well written paper aims to describe how researchers can prepare their data to be more FAIR, taking the specific example of plant data. The paper lays out a set of pragmatic guidelines to move the community closer to FAIRer data and good data management in general and concludes with an assessment of their test data via a number of recent FAIR data assessment frameworks.

There are many good pieces of advice in the paper and all reasoning is well explained. However, the focus on spreadsheet data, while understood, given spreadsheets are, in the words of the paper, 'the tool par excellence', does limit the scope somewhat, as some of the guidelines could be expanded to other types of data. However, given this focus, the paper would benefit from having more examples, explanations and

Powered by Editorial Manager® and ProduXion Manager® from Aries Systems Corporation

recommendations of specific controlled vocabularies or reporting guidelines to follow (e.g. Why was ODAM chosen? Which CVs were chosen, are the CVs FAIR?)

Answer: In this paper, we wanted to focus on the benefit of FAIRification integrated into the data management process based on good practices, and not on FAIRification in general. In addition, as the title indicates, we have illustrated it from tables of experimental data by proposing a specific approach to this type of data. This document being a commentary, i.e. a short paper (1500 words maximum, 10 references maximum) did not allow us to develop all the points of FAIRification.
Concerning FAIRification in general and more specifically on the respect of the FAIR principles, we rely on a very recent paper (Jacobsen et al 2020, given in the references) which gives all the necessary information.

Nevertheless, concerning the points that you mention, we have added elements or precision allowing us to answer them in part because of the limit imposed on the size of the text, as follow:
-Why was ODAM chosen? see lines 67-75, core text.
-Which CV were chosen? are the CVs FAIR? see lines 243-246, figure 1 legend

I have two further specific points:

1. The authors choose (on line 90) the Frictionless Data JSON specifications as their interoperability standard of choice. This choice should be explained as many other interoperability standards are also available. This explanation could also touch on the use of metadata to place the dataset into context, a key factor to make the data FAIR, which is mentioned in the Frictionless Data specification but not in the main text.

Answer: Indeed, we had somewhat omitted this aspect in the first version of our manuscript. This is why we have remedied it in the figure 2 legend, lines 283-290. In addition, we have explicitly mentioned the specification in the main text. See line 97.

2. One issue that hasn't been covered in the Publication of data section (line 98-115) is that of data licensing. This is an important issue in FAIR and should, in my opinion, be mentioned in this section.

Answer: This is one of the aspects that would require further development but would be too much for this comment. Nevertheless, we have briefly mentioned this important point. See lines 151-153

For clarity, I would suggest the following additions to the text:

1.Line 90 - state the open, interoperability standard, rather than just the reference (so readers don't have to go to the reference list to find the name of the standard).

Answer: Right! We have mentioned the name of the standard in the main text. See line 97.

2. In general, the standards mentioned in the figures should also be mentioned in the text, as the figures seem slightly disconnected at present. For example, the use of ODAM in figure 1. ODAM isn't mentioned in the main text but is integral to the work.

Answer: Right! We have mentioned ODAM in the main text. See line 73.

There are a few typographical errors:

1. line 39 'e.g. *the* European commission'
Answer: Ok, corrected

2. line 59-60 'Thus, a tool *enabling the automatic combination of* data sets'
Answer: As the text has changed, this is no longer applicable. But indeed, we have paid attention to the use of these verbs in English (allow X to, enable X to) in this paper. Thank you.

	3. line 75 'because *they are* essentially using'<br>Answer: As the text has changed, this is no longer applicable.<br><br>4. line 122 '2. *Researchers have* the best control'<br>Answer: Ok, corrected<br><br>The paper may also benefit from the addition of a reference on line 103 - 'Journal editors and reviewers are increasingly recommending that all data, complete and non-synthetic, generated during the course of the study be made available' - it would be good to provide a reference as evidence of this (I don't doubt the veracity, I just think it should be accompanied by a reference).<br><br>Answer: We looked for a reference that could support this opinion (based on our own observations when submitting papers), but found nothing satisfactory. Ideally, it could have been a survey of publishers and reviewers. The only one we found does not directly concern this point (see below). So we decided to delete this sentence, which saved us a few extra words (valuable in a short paper!).<br><br>Survey on open peer review: Attitudes and experience amongst editors, authors and reviewers<br>https://journals.plos.org/plosone/article?id=10.1371/journal.pone.0189311<br><br>I'd like to conclude by congratulating the authors. I particularly enjoyed and agree with the statement that starts on line 94 - 'So, in our approach data FAIRification is closely related to data management, avoiding a retroactive process that would require more time, costs and computer skills'.<br><br>This paper has many merits, not least as an example of how FAIR can and should be put into practice.<br><br>Answer: Thank you for your encouragement, very appreciated!
**Additional Information:**	
**Question**	**Response**
Are you submitting this manuscript to a special series or article collection?	No
**Experimental design and statistics**<br><br>Full details of the experimental design and statistical methods used should be given in the Methods section, as detailed in our Minimum Standards Reporting Checklist. Information essential to interpreting the data presented should be made available in the figure legends.<br><br>Have you included all the information requested in your manuscript?	No
If not, please give reasons for any omissions below.	The data mentioned in this paper have already been published in several articles. This paper does not concern statistical processing about data but mainly their management.

as follow-up to "**Experimental design and statistics**<br><br>Full details of the experimental design and statistical methods used should be given in the Methods section, as detailed in our Minimum Standards Reporting Checklist. Information essential to interpreting the data presented should be made available in the figure legends.<br><br>Have you included all the information requested in your manuscript?<br><br>"	
**Resources**<br><br>A description of all resources used, including antibodies, cell lines, animals and software tools, with enough information to allow them to be uniquely identified, should be included in the Methods section. Authors are strongly encouraged to cite Research Resource Identifiers (RRIDs) for antibodies, model organisms and tools, where possible.<br><br>Have you included the information requested as detailed in our Minimum Standards Reporting Checklist?	Yes
**Availability of data and materials**<br><br>All datasets and code on which the conclusions of the paper rely must be either included in your submission or deposited in publicly available repositories (where available and ethically appropriate), referencing such data using a unique identifier in the references and in the "Availability of Data and Materials" section of your manuscript.<br><br>Have you have met the above	Yes

requirement as detailed in our Minimum Standards Reporting Checklist?	

The revised version of the manuscript
Click here to access/download;Manuscript;ODAM-FAIR_GigaScience_v2.docx

Towards FAIRification of experimental data tables in life sciences: a pragmatic approach

Daniel Jacob[1,2*], Romain David[3,4], Sophie Aubin[5], Yves Gibon[1,2]

[*]Corresponding author

Institutional addresses:

[1] INRAE, Université de Bordeaux, UMR BFP, 71 av E Bourlaux, 33140 Villenave d'Ornon, France

[2] PMB-Metabolome, INRAE, 2018. Bordeaux Metabolome Facility, MetaboHUB, 33140 Villenave d'Ornon, France. doi: 10.15454/1.5572412770331912E12

[3] INRAE, Montpellier SupAgro, Université de Montpellier, UMR MISTEA, 2, place Pierre Viala, 34060 Montpellier Cedex 2, France

[4] European Research Infrastructure on Highly Pathogenic Agents (ERINHA-AISBL), 101 rue de Tolbiac, 75013 Paris, France

[5] INRAE, DipSO, 42 rue Georges Morel, 49070 Beaucouzé, France,

Email addresses, ORCID :

DJ: daniel.jacob@inrae.fr, ORCID: 0000-0002-6687-7169

RD: romain.david@erinha.eu, ORCID: 0000-0003-4073-7456

SA: sophie.aubin@inrae.fr ORCID: 0000-0003-4805-8220

YG: yves.gibon@inrae.fr ORCID: 0000-0001-8161-1089

Abstract

Making data compliant with the FAIR Data principles (Findable, Accessible, Interoperable, Reusable) is still a challenge for many researchers, who are not sure which criteria should be met first and how. Illustrated from experimental data tables associated with a Design of Experiments, we propose an approach that can serve as a model for research data management that allows researchers to disseminate their data by satisfying the main FAIR criteria without insurmountable efforts. More importantly, this approach aims to facilitate the FAIRification process by providing researchers with tools to improve their data management practices.

Keywords: Research Data Management; FAIR Data principles; FAIR assessment; experimental data tables

Background

The publication of research data according to the FAIR principles [1] has become a major challenge with the aim of integrating them into the overall research process (e.g. the European commission explicitly mentions FAIR principles as a mandatory reference [2]). However, implementing these principles is not so easy and requires changes in data management practices. According to Jacobsen et al [3], the FAIR principles can be seen as a consolidation of good data management practices to extend management to the notion of machine reuse of data. Thus, it seems appropriate to use virtuous principles as far upstream as possible from the data rather than trying to comply with FAIR principles downstream. This is the starting point of the approach we propose in order to integrate these principles into the practices of researchers.

Set the scene

Let's take a concrete example from the plant biology domain. A study on the metabolism of tomato fruits [4] involved growing several hundred tomato plants in greenhouses. This multifactorial experiment (stages of development and type of treatment applied to each group of plants) generated a dozen large experimental data tables. Part of the data was acquired in the greenhouse manually using spreadsheets. While another part of the data comes from biochemical and metabolomics analyses, carried out on the thousand samples taken and returned in the form of data tables weeks or even months later.

The use of spreadsheets is therefore central here, as it is a tool that researchers master very well. However, manual handling is required to link together experimental data tables from several analytical techniques, according to samples. Such repeated data handling, a potential source of errors, can compromise the consistency of data, which must be managed throughout the study by ensuring that each analysis is well linked to its sample. We needed to review our data management practices. The question remained as to how to motivate and convince researchers to change their practice a little.

To promote good practices, provide services

Efforts have been undertaken for several years to propose format standards in order to be able to disseminate its data according to FAIR principles and in particular experimental data tables (e.g. ISA-TAB [5]). In our approach, the emphasis has been mainly on the integration of FAIR principles from the beginning of the data's life, i.e. as soon as they are acquired. Thus, we have focused on the structural metadata related to the experimental data in the spreadsheets, i.e. how they are organized so that we can more easily exploit them. The objective of our approach called ODAM (Open Data for Access and Mining) is to make this upstream capture an advantage to facilitate data analysis and therefore an incentive to perform this metadata capture. By relying on the tool that researchers know best, i.e. spreadsheets, it was nevertheless necessary to remedy its drawbacks and in particular the lack of constraints in the structuring of data. In this perspective, we propose a data structuring similar to data dictionaries that is easy to implement by the researchers themselves (**Figure 1, Additional file 1**). Structural metadata (e.g. links between data tables) are described, together with unambiguous definitions of all internal elements (e.g. column definitions along with their semantic definition), through links to accessible definitions, such as community-approved ontologies where possible, as recommended by Jacobsen et al [3]. But for good practices to be adopted, researchers must take advantage of them. Thus, we propose tools that greatly facilitate the combination and merging of data sets according to a common attribute (identifiers) allowing the analysis of several types of variables according to different parameters without any tedious manipulation of the data, offering researchers a very appreciable time saving by avoiding repetitive and tedious tasks. Besides, depending on the needs and skills of researchers, data can be used in a wide variety of ways (**Figure 2**).

The advantage of this approach is manifold. It allows the data to be structured in such a way that the researcher i) can proceed step by step as the data become available and ii) can easily exploit it with tools immediately afterwards. In doing so, FAIRification of data is carried out in order to handle data more efficiently and not just to publish it. Thus, it is the FAIRification of data that is integrated by design into the data processing workflow. So, in our approach data FAIRification is

closely related to data management, avoiding a retroactive process that would require more time, costs and computer skills [6,7]. In addition, from the structured metadata it becomes possible to convert them directly into a standard format. In our case, we chose the "Frictionless datapackage" (https://frictionlessdata.io/) a community, open, interoperability standard (**Figure 2 & 3**). Slightly adapted to our needs, for example by specifying the category of each attribute, the data can thus be disseminated according to an open schema that greatly facilitates the reuse of data by machines. The choice of the data repository to disseminate its data formatted in this way, is quite open because a clear separation is established between structural metadata on the one hand, and descriptive metadata depending on the type of repository, on the other hand. However, on the basis of metadata files this does not prevent the development of converters to other formats such as the complex data model (e.g. ISA-TAB [5]) in order to include the data in existing standards-compliant data infrastructures (e.g. SEEK data management platform [8]).

Publication of data

When it comes to publishing their results, researchers usually provide the minimum required to support their claims [9]. This generally results in a loss of data (quantitative aspect) and information (qualitative aspect) compared to the totality of the data acquired during the study. We believe that our approach should facilitate the dissemination of the complete dataset because the work has been done upstream, and that when needed the data can be deposited in an appropriate repository quickly enough without having to do data archaeology, while at the same time meeting the essential criteria of the FAIR principles is guaranteed (**Additional files 2,3 and 4**). In addition, the expected benefits are numerous, including exploiting the full potential of the data sets, improving the reproducibility and reliability of the data, but also increasing visibility and citation due to the reuse of the data by both humans and machines.

Furthermore, a lever of motivation would be the recognition of this effort to publish data according to the FAIR principles. Unfortunately, researchers who devote time and expertise to activities like data curation are not currently rewarded by traditional career progression metrics. We believe

that this should change in the future, and crediting and rewarding mechanisms are the subject of the Research Data Alliance SHAring Rewards and Credit Interest Group [10].

Summary of the proposed approach and beyond

1. To provide researchers with services that are truly useful, time-saving and efficient. Care must also be taken not to deprive them of their know-how or trap them in turnkey solutions that prevent any opportunity of testing several hypotheses or scenarios. Rather, we need to open the data to a whole ecosystem of software possibilities.
2. Since researchers have the best control and understanding of their data, they are in the best position to annotate it. It is therefore advisable to help them as much as possible in this process, by offering them protocols and methods adapted to their IT skills, an area that is not their core business. In particular, vocabulary dictionaries corresponding to their domain and frequently used should be provided in order to standardize annotations as much as possible.
3. Behind a dataset, there is often an involved team with a wide range of skill levels. We must take into account their way of working, their work habits; so instead of wanting to change their habits completely, we must rather adapt them in a way that is beneficial to them, and make them actors in the data FAIRification process. Probably the best FAIR training is the one based on their data. But it is necessary to capitalize on good practices in written protocols, and referenced into data management plans.
4. Researchers must be involved in the data FAIRification process by providing them with tools for assessing their practices so that they can progressively improve them in stages, having properly integrated each of the criteria implemented. Especially, these assessment tools should highlight all the steps where small actions can significantly improve the FAIRification of the data. They will thus be more inclined to integrate them into their practice with full knowledge of the facts.

5. Concerning the data provenance: not only the authors, but above all the context, the methods of data acquisition and processing are crucial information for a good reuse. Unfortunately, this aspect is somewhat neglected, if not absent. An effort still needs to be made in this direction in order to sensitize and motivate data producers. Finally, it should be mentioned that the license of the data must be appropriate for the reuse of the data.

Declarations

Ethics approval and consent to participate

Not applicable.

Consent for publication

Not applicable.

Availability of data and material

All information, documents, data and software concerning ODAM are accessible from Github [10]

Competing interests

The authors declare that they have no competing interests

Funding

DJ, RD and YG were partly supported by the PHENOME-EMPHASIS project funded by the French National Research Agency (ANR-11-INBS-0012). DJ and YG were also supported by the FRIMOUSS project funded by the French National Research Agency (ANR-15-CE20-0009-01). RD was also partly supported by the EPPN2020 project (H2020 grant N°731013), the EOSC-Life european program (grant agreement N°824087). SA was partly supported by the FooSIN project funded by the French National Research Agency (ANR- 19- DATA- 0019-01). All authors were also partly funded by the French National Research Institute for Agriculture, Food and the Environment (INRAE). The FRIM1 dataset came from research supported by the Eranet Erasysbio+ FRIM project funded by the French National Research Agency (ANR-09-SYSB-003) and the MetaboHUB project funded by the French National Research Agency (ANR-11-INBS-0010).

Author contributions

conceptualization: D.J; data curation: D.J; funding acquisition: Y.G; methodology: D.J, R.D; software: D.J; writing—original draft: D.J, R.D; writing—review and editing: D.J, R.D, S.A, Y.G. All authors read and approved the final manuscript.

Acknowledgements

We thank Catherine Deborde (PMB-Metabolome, INRAE, MetaboHUB) for advice on the manuscript and for constructive reviews.

References

1. Wilkinson MD, Dumontier M, Aalbersberg IJJ, Appleton G, Axton M, Baak A, et al. The FAIR Guiding Principles for scientific data management and stewardship. Sci Data. 2016;3:160018. doi:10.1038/sdata.2016.18.
2. European Commission Directorate General for Research and Innovation (2018) Turning FAIR into reality, Final Report and Action Plan from the European Commission Expert Group on FAIR Data, https://ec.europa.eu/info/publications/turning-fair-reality_en [Accessed June 04, 2020]
3. Jacobsen A, de Miranda Azevedo R, Juty N, Batista D, Coles S, Cornet R et al (2020) Data Intelligence 2: 1-2, 10-29 doi:10.1162/dint_r_00024
4. Bénard C, Biais B, Ballias P, Beauvoi B, Bernillon S, Cabasson C et al (2018), FRIM - Fruit Integrative Modelling, doi.org/10.15454/95JUTK, Portail Data INRAE, V3
5. Sansone, S., Rocca-Serra, P., Field, D. et al (2012), Toward interoperable bioscience data, Nat Genet 44, 121–126, doi:10.1038/ng.1054
6. Rocca-Serra P and Sansone SA (2019) Experiment design driven FAIRification of omics data matrices, an exemplar, Scientific Data volume 6, 271 doi:10.1038/s41597-019-0286-0
7. European Commission, Directorate-General for Research and Innovation (2018), Cost-Benefit analysis for FAIR research data - Cost of not having FAIR research data, https://op.europa.eu/en/publication-detail/-/publication/d375368c-1a0a-11e9-8d04-01aa75ed71a1 [Accessed June 04, 2020]

8. Wolstencroft K., Owen S., Krebs O., Nguyen Q. et all (2015) SEEK: A systems biology data and model management platform, BMC Systems Biology 9:33, doi:10.1186/s12918-015-0174-y

9. Leonelli S, Smirnoff N, Moor J (2013) Making open data work for plant scientists, Journal of Experimental Botany, pp. 4109–4117, doi:10.1093/jxb/ert273

10. David R, Mabile L, Specht A, Stryeck S, Thomsen M, Yahia M et al. (2020) FAIRness Literacy: the Achilles' Heel of applying FAIR Principles. Hal-02483307 https://hal.inrae.fr/hal-02483307

Figures

Figure 1: ODAM (Open Data for Access and Mining) is an Experiment Data Table Management System (EDTMS) based on good data management practices concerning data structuring and the description of structural metadata. Indeed, the strong point of the approach is to define metadata in depth, i.e. at the level of the data itself (i.e. metadata at column-level such as factors, variables...) and not only as a "hat" on the data set. Thus, having structural metadata allows datasets to achieve a higher level of interoperability and greatly facilitates functional interconnection and analysis in a broader context. (**A**) To simplify, we have considered here the first two tables of data from the experiment, namely the individuals (*plants.txt*) followed by the samples (*samples.txt*). The data must be well organized i.e. each variable forms a column, each observation forms a row, and each table is relative to an entity i.e. the same type of observational unit (plants, samples, ...), and a file must contain only one data table. Since all experimental data tables were generated in an experiment associated with a design of experiment, the data tables were acquired sequentially as the experiment progresses. A link must exist between each of them, generally defined by identifiers. In our example, each sample is linked to the plant from which it comes from. (**B**) Furthermore, whatever the type of experiment, it requires a design of experiment involving individuals, samples or whatever, as the main objects of study and producing several tables of experimental data. It also involves the observation of dependent variables resulting from the effects of certain controlled independent variables (*factors*). In addition, the objects of study usually have an *identifier* for each one, and the variables can be *quantitative* or *qualitative*. Thus,

each of the columns within a table (*attributes*) can be associated with one of the four categories: *identifier, factor, quantitative, qualitative.* By associating a category to each column, this greatly facilitate subsequent statistical analyses by the machines. All structural metadata can be grouped in two specific files. (**C**) The first metadata file associates to each data table (*subset*) a key concept corresponding to the main entity of the data table. It also defines for each table the link with the table from which it comes from (magenta arrows). These links can be interpreted as "is obtained from". (**D**) The second metadata file annotates each attribute (concept/variable) with minimal but relevant metadata, such as: its category defined above, its description with its unit, the data type. In each of these two files (entities and attributes), it is possible to annotate each of the terms with unambiguous definitions (CV terms) through links to accessible (standard) definitions based on ontologies. The choice of ontologies is very domain-specific but nevertheless it should preferably be based on those that follow the FAIR principles [r1]. In the case of the FRIM experiment, we mainly used AgroPortal [r2] and especially its "annotator" module made efficient thanks to the alignment of ontologies. Since these ontological terms are not essential for statistical analysis, they can be omitted up to the publication stage. It should be noted that tools for adding ontology terms to Excel spreadsheets are still being developed for ODAM software suite to facilitate this tedious task [r3]. Some tools such as RightField [r4], ISA-Tools [r5] or Swate [r6] offer interesting approaches and will be for sure good inspiration sources. Knowing that ontological terms are essential mainly for data dissemination, a connection with the ISA-TAB format for instance would make it possible to benefit from the tools already available for this type of task. In any case, established mainly by and for the scientists who produced the data, this structural metadata will later allow non-expert users to explore and visualize the data, thus offering a better guarantee of correct (re)use by those who did not produce them. See Data Preparation Protocol for ODAM Compliance for more details (**Additional file 1**).

r1. A. Jacobsen et al (2020) Data Intelligence, doi:10-29 doi:10.1162/dint_r_00024
r2. C. Jonquet et al (2018) Computers and Electronics in Agriculture, doi:10.1016/j.compag.2017.10.012
r3. https://inrae.github.io/ODAM/todo last accessed: 2020-11-05
r4. https://rightfield.org.uk/ last accessed: 2020-10-15
r5. S. Sansone et al (2012) Nat Genet 44, 121–126, doi:10.1038/ng.1054
r6. https://github.com/nfdi4plants/Swate last accessed: 2020-10-15

Figure 2: ODAM software suite: In light blue (promote <-> provide) the engine of the approach, in purple the data and metadata provided by the user, in dark blue the activities related to the life cycle of the data. The whole process is implemented primarily to make better use of its data before its dissemination. The ODAM software embeds an API (Application Programming Interface) layer that allows interoperability between the different tables and the applications that will be able to use them. With the help of this layer, it opens up a whole ecosystem of potential applications, depending on your needs but also on your skills in the proposed tools. From the set of data files (which are non-combined tables, each corresponding to a particular observational unit that we name an entity), the user can: 1) Visualize the data associated with their metadata according to several criteria and in a completely interactive way with the help of the data explorer. 2) Export in tabular form subsets selected according to his criteria with combined, merged data. 3) Build and test his models more easily using a scripting language such as R, which allows it to repeat different scenarios according to a variety of parameters. All this is made possible thanks to the category as controlled vocabulary associated with each column, which facilitates statistical analysis by both humans and machines. Moreover, the first available data can be exploited as soon as the corresponding metadata have been captured without waiting until all the data are available. The benefit of this approach is that the "life of the data" is integrated into the scientific research process, according to good data management practices that meet the essential FAIR criteria. Then, distributed data is enriched by associating a structural metadata file called datapackage [r1], a simple container which serves as metadata aggregator based on JSON schema specifications, an open, community-based interoperability standard. This compact and hierarchically structured format proved to be suitable for integrating all of our structural metadata, thus placing the dataset in its experimental context, a key factor in making the data FAIR. Data generation according to this open schema is included in the proposed tools and does not require additional effort for the researcher. The definition of an explicit schema for structural metadata thus enables machines to better interpret the data for reuse. Indeed, exporting this metadata in datapackage format offers a great flexibility of use data via scripting languages such as R and

Python on the basis of existing packages. Besides, this type of format allows a great variety in the choice of data repository as a distinct separation is established between structural metadata described in the datapackage format on the one hand, and descriptive metadata depending on the type of repository on the other hand. Preferably the chosen data repository should offer the ability to query and retrieve data using an API that conforms to the OpenAPI specification [r2] and that meet the essential criteria of the FAIR principles. For example, the following data repositories registered in re3data.org [r3] can be cited without being exhaustive: Dataverse [r4], Dryad [r5], FAIRDOMHub [r6], FigShare [r7], Zenodo [r8]. Finaly, the FAIRification can be considered from two points of view: 1/ It is linked to the data life cycle by the annotations and curations made on the data themselves, and to the quantity and quality of the information associated with the data (protocols, publications, keywords, ...), 2/ it can also be considered from the point of view of its data management practices, which must improve over time, which is precisely what the FAIR assessment grids attempt to measure, and more particularly the reproducibility and reusability of the data. See ODAM Deployment and User's Guide for more details [r9].

r1. https://frictionlessdata.io/ last accessed: 2020-10-15
r2. http://spec.openapis.org/oas/v3.0.3 last accessed: 2020-10-15
r3. http://re3data.org/ last accessed: 2020-10-15
r4. https://dataverse.org/ last accessed: 2020-10-15
r5. https://datadryad.org/ last accessed: 2020-10-15
r6. K. Wolstencroft et al (2017) Nucleic Acids Res, DOI : 10.1093/nar/gkw1032
r7. https://figshare.com/ last accessed: 2020-10-15
r8. https://zenodo.org/ last accessed: 2020-10-15
r9. https://inrae.github.io/ODAM/ last accessed: 2020-11-05

Figure 3: Interconnection of the different elements of the FRIM dataset from the Data INRAE repository [r1] as a hub (based on Dataverse), a data repository that complies with the JSON-LD standard. Distributed data is enriched by associating a structural metadata file called datapackage [r2], a simple container format based on JSON schema specifications, an open, community-based interoperability standard. Schematically, the role of the data repository mainly ensures the "Findable" and "Accessible" criteria of the FAIR principles from the descriptive metadata, whereas the datapackage mainly ensures the "Interoperable" and "Reusable" criteria from the structural metadata, even if these roles are not exclusive. To be compliant with the FAIR principles, not all

data, documents, workflows and other tools need to be located in a single system, but from a central repository, it is the set of links that constitutes the true information management system. It must be able to be traversed by a human being as well as by machines. By relying on explicit schemas (JSON-LD, JSON Schema) for both metadata and data, it becomes possible to reuse the data without friction, both by humans and machines. The use of spreadsheets greatly facilitates the annotation of data with metadata by the data producers themselves. Thus, this is technology, however powerful, that becomes part of the practices of non-experts in the domain, not the other way around. In addition, this further enhances the FAIR criteria, especially the reuse and interoperability criteria. To evaluate the level of the FAIRness, we used three FAIR grids, very different from each other. The first one, the OZONOME 5-star data ranking tool [r3], aims to perform an evaluation based on the FAIR principles as defined by Willkinson et al [r4]. The main result is an overall rating, indicating the overall fairness of the data set. The other two grids are dedicated to a more refined assessment. The Fair Data Maturity Model (FDMM) document [r5] describes a maturity model for the FAIR assessment with indicators, priorities and assessment methods, which are useful for standardizing assessment approaches in order to allow comparison of their results. Whereas the FAIR SHARC (SHAring Rewards and Credit) [r6] document allows the fairness of projects and associated human processes to be assessed, either by external evaluators or by the researchers themselves. Therefore, these grids cannot be compared with each other, but rather complement each other. Overall, the FAIRness of our dataset using the ODAM+Dataverse combination is of a good standard. However, to achieve complete FAIRification, we need to move towards semantic web approaches [r7]. By relying on explicit data schemas, the effort to climb this mountain can be envisaged with less fear.

r1. Institut National de Recherche pour l'Agriculture, l'Alimentation et l'Environnement. (2018). Data INRAE. DOI: 10.14758/9T8G-WJ20

r2. https://frictionlessdata.io/ last accessed: 2020-10-15

r3. https://confluence.csiro.au/display/OZNOME/Data+ratings last accessed: 2020-10-15

r4. MD. Willkinson et al (2016) Sci Data. DOI: 10.1038/sdata.2016.18

r5. https://www.rd-alliance.org/groups/fair-data-maturity-model-wg last accessed: 2020-10-15

r6. R. David et al (2020) Data Science. Journal. DOI: 10.5334/dsj-2020-032

r7. https://inrae.github.io/ODAM/todo last accessed: 2020-11-05

Additional files

Additional file 1. Data Preparation Protocol for ODAM Compliance. The purpose of this protocol is to describe all the steps involved in collecting, preparing and annotating the data from an experiment associated with an experimental design (DoE) that will then allow the user to benefit from the services offered by ODAM.

Additional file 2. FAIR evaluation of the FRIM1 dataset according to the 5 ★ Data Rating Tool grid. It aims to perform an evaluation based on the FAIR principles as defined by Willkinson et al. [1]. The main result is an overall rating, indicating the overall fairness of the data set.

Additional file 3. FAIR assessment of the FRIM1 dataset according to the FDMM (FAIR Data Maturity Model) grid. This document describes a maturity model for the FAIR assessment with indicators, priorities and assessment methods, which are useful for standardizing assessment approaches in order to allow comparison of their results.

Additional file 4. FAIR assessment of the FRIM1 dataset according to the SHARC (Sharing Rewards and Credit) grid. This document allows the fairness of projects and associated human processes to be assessed, either by external evaluators or by the researchers themselves.

Manuscript with the follow-up of the changes made from the previous version

Click here to access/download;Manuscript;ODAM-FAIR_GigaScience_v1_revised.docx

Towards FAIRification of experimental data tables in life sciences: a pragmatic approach

Daniel Jacob[1,2*], Romain David[3,4], Sophie Aubin[5] , Yves Gibon[1,2]

[*]Corresponding author

Institutional addresses:

[1] INRAE, Université de Bordeaux, UMR BFP, 71 av E Bourlaux, 33140 Villenave d'Ornon, France

[2] PMB-Metabolome, INRAE, 2018. Bordeaux Metabolome Facility, MetaboHUB, 33140 Villenave d'Ornon, France. doi: 10.15454/1.5572412770331912E12

[3] INRAE, Montpellier SupAgro, Université de Montpellier, UMR MISTEA, 2, place Pierre Viala, 34060 Montpellier Cedex 2, France

[4] European Research Infrastructure on Highly Pathogenic Agents (ERINHA-AISBL), 101 rue de Tolbiac, 75013 Paris, France

[5] INRAE, DipSO, 42 rue Georges Morel, 49070 Beaucouzé, France,

Email addresses, ORCID :

DJ: daniel.jacob@inrae.fr, ORCID: 0000-0002-6687-7169

RD: romain.david@erinha.eu, ORCID: 0000-0003-4073-7456

SA: sophie.aubin@inrae.fr ORCID: 0000-0003-4805-8220

YG: yves.gibon@inrae.fr ORCID: 0000-0001-8161-1089

Abstract

Making data compliant with the FAIR Data principles (Findable, Accessible, Interoperable, Reusable) is still a challenge for many researchers, who are not sure which criteria should be met first and how. Illustrated from experimental data tables associated with a Design of Experiments, we propose an approach that can serve as a model for ~~a~~ research data management that allows researchers to disseminate their data by satisfying the main FAIR criteria without insurmountable efforts. More importantly, this approach aims to facilitate the FAIRification process by providing researchers with tools to improve their data management practices.

Keywords: Research Data Management; FAIR Data principles; FAIR assessment; experimental data tables

Background

The publication of research data according to the FAIR principles [1] has become a major challenge with the aim of integrating them into the overall research process (e.g. the European commission explicitly mentions FAIR principles as a mandatory reference [2]). However, implementing these principles is not so easy and requires changes in data management practices. According to Jacobsen et al [3], the FAIR principles can be seen as a consolidation of good data management practices to extend management to the notion of machine reuse of data. Thus, it seems appropriate to use virtuous principles as far upstream as possible from the data rather than trying to comply with FAIR principles downstream. This is the starting point of the approach we propose in order to integrate these principles into the practices of researchers.

Set the scene

Let's take a concrete example from the plant biology domain. A study on the metabolism of tomato fruits [4] involved growing several hundred tomato plants in greenhouses. This multifactorial experiment (stages of development and type of treatment applied to each group of plants) generated a dozen large experimental data tables. Part of the data was acquired in the greenhouse manually using spreadsheets. While another part of the data comes from biochemical and metabolomics analyses, carried out on the thousand samples taken and returned in the form of data tables weeks or even months later.

The use of spreadsheets is therefore central here, being the tool par excellence for researchers. However, many manual handling is required to link together experimental data tables from several analytical techniques, according to samples. Such repeated data handling, a potential source of errors, can compromise the consistency of data, which must be managed throughout the study by ensuring that each analysis is well linked to its sample. ~~In addition, this study involved several partners who wanted to access the data as soon as possible. We needed to review our data~~

management practices. The question remained as to how to motivate and convince researchers to change their practice a little.

To promote good practices, provide services

Efforts have been undertaken for several years to propose format standards in order to be able to disseminate its data according to FAIR principles and in particular experimental data tables (e.g. ISA-TAB [5]). In our approach, the emphasis has been mainly on the integration of FAIR principles from the beginning of the data's life, i.e. as soon as they are acquired. Thus, we have focused on the structural metadata related to the experimental data in the spreadsheets, i.e. how they are organized so that we can more easily exploit them. The objective of our approach called ODAM (Open Data for Access and Mining) is to make this upstream capture an advantage to facilitate data analysis and therefore an incentive to perform this metadata capture. By relying on the tool that researchers know best, i.e. spreadsheets, it was nevertheless necessary to remedy its drawbacks and in particular the lack of constraints in the structuring of data. In this perspective, we propose a data structuring similar to data dictionaries that is easy to implement by the researchers themselves (**Figure 1, Additional file 1**). Structural metadata (e.g. links between data tables) are described, together with unambiguous definitions of all internal elements (e.g. column definitions along with their semantic definition), through links to accessible definitions, such as community-approved ontologies where possible, as recommended by Jacobsen et al [3]. But for good practices to be adopted, researchers must take advantage of them. Thus, we propose tools that greatly facilitate the combination and merging of data sets according to a common attribute (identifiers) allowing the analysis of several types of variables according to different parameters without any tedious manipulation of the data, offering researchers a very appreciable time saving by avoiding repetitive and tedious tasks. Besides, depending on the needs and skills of researchers, data can be used in a wide variety of ways (**Figure 2**).

The advantage of this approach is manifold. It allows the data to be structured in such a way that the researcher i) can proceed step by step as the data become available and ii) can easily exploit it with tools immediately afterwards. In doing so, FAIRification of data is carried out in order to handle data more efficiently and not just to publish it. Thus, it is the FAIRification of data that is integrated by design into the data processing workflow. So, in our approach data FAIRification is closely related to data management, avoiding a retroactive process that would require more time, costs and computer skills [6,7]. In addition, from the structured metadata it becomes possible to convert them directly into a standard format. In our case, we chose the "Frictionless datapackage" (https://frictionlessdata.io/) a community, open, interoperability standard (**Figure 2 & 3**). Slightly adapted to our needs, for example by specifying the category of each attribute, the data can thus be disseminated according to an open schema that greatly facilitates the reuse of data by machines. The choice of the data repository to disseminate its data formatted in this way, is quite open because a clear separation is established between structural metadata on the one hand, and descriptive metadata depending on the type of repository, on the other hand. However, on the basis of metadata files this does not prevent the development of converters to other formats such as the complex data model (e.g. ISA-TAB [5]) in order to include the data in existing standards-compliant data infrastructures (e.g. SEEK data management platform [8]).To bring about a change in habits, an improvement in the gains/efforts ratio can act as a motivational lever. This is precisely the basis of our approach: to provide tools and services that are truly useful to researchers in order to get them to adopt good practices. Thus, a tool allowing to automatically combine data sets according to a common attribute (identifiers) would be easily adopted because it would allow several types of variables to be analyzed according to different parameters without any tedious data handling. Now, it is very likely that the spreadsheet will continue to be used despite its drawbacks, in particular the lack of constraint on information structure that, paradoxically, makes it so popular. Rather, an approach is needed that largely avoids such drawbacks. In this perspective, we propose a data structuring similar to data dictionaries that is

easy to implement by the researchers themselves, because essentially using a spreadsheet (**Figure 1**). Structural metadata (*e.g.* links between data tables) are described, together with unambiguous definitions of all internal elements (e.g. column definitions along with their semantic definition), through links to accessible definitions, such as community-approved ontologies where possible, as recommended by Jacobsen et al [3]. The change in practice consists mainly in doing this structuring work upstream of any data processing, knowing that from this structuring, the researchers can thus have tools at their disposal to avoid very time-consuming, repetitive and tedious tasks later on. Better still, it opens up data on a whole ecosystem of potential applications, according to their needs and skills (**Figure 2**).

The advantage of this approach is manifold. It allows the data to be structured in such a way that the researcher *i*) can proceed step by step as the data become available and *ii*) can easily exploit it with tools immediately afterwards. In doing so, the researcher performs a data FAIRification in order to handle it more efficiently and not only for publishing it. Thus, it is the FAIRification of data that is integrated by design into the data processing workflow. In addition, from the structured metadata it becomes possible to convert them directly into a standard format. In our case, we used a data format based on a community, open, interoperability standard [5]. Slightly adapted to our needs *e.g.* by specifying the category of each attribute, it now enables to disseminate the data according to an open schema that greatly facilitates the reuse of the data by machines (**Figure 3**). Data generation according to this open schema is included in the proposed tools and does not require additional effort for the researcher. So, in our approach data FAIRification is closely related to data management, avoiding a retroactive process that would require more time, costs and computer skills [6,7].

Publication of data

When it comes to publishing their results, researchers usually provide the minimum required to support their claims [8]. This generally results in a loss of data (quantitative aspect) and information (qualitative aspect) compared to the totality of the data acquired during the study. ~~However, journal editors and reviewers are increasingly recommending that all data, complete and non-synthetic, generated during the course of the study be made available.~~ We believe that our approach should facilitate this dissemination of the complete dataset because the work has been done upstream, and that when needed the data can be deposited in an appropriate repository quickly enough without having to do data archaeology, while at the same time meeting the essential criteria of the FAIR principles is guaranteed (**Additional files 1,2 and 3**). In addition, the expected benefits are numerous, including exploiting the full potential of the data sets, improving the reproducibility and reliability of the data, but also increasing visibility and citation due to the reuse of the data by both humans and machines.

Furthermore, a lever of motivation would be the recognition of this effort to publish data according to the FAIR principles. Unfortunately, researchers who devote time and expertise to activities like data curation are not currently rewarded by traditional career progression metrics. We believe that this should change in the future, and crediting and rewarding mechanisms are the subject of the Research Data Alliance SHAring Rewards and Credit Interest Group [9].

Summary of the proposed approach and beyond

1. To provide researchers with services that are truly useful, time-saving and efficient. Care must also be taken not to deprive them of their know-how or trap them in turnkey solutions that prevent any opportunity of testing several hypotheses or scenarios. Rather, we need to open the data to a whole ecosystem of software possibilities.
2. Since researchers have the best control and understanding of their data, they are in the best position to annotate it. It is therefore advisable to help them as much as possible in

this process, by offering them protocols and methods adapted to their IT skills, an area that is not their core business. In particular, vocabulary dictionaries corresponding to their domain and frequently used should be provided in order to standardize annotations as much as possible.

2. ~~They are the ones who have the best control and understanding over their data, so they are in the best position to annotate it. On the other hand, it is necessary to assist them as much as possible in this process, by proposing protocols and methods adapted to their computer skills, a domain which is not their core expertise. In particular, dictionaries of vocabulary corresponding to their field and frequently used should be provided in order to standardize the annotations as much as possible.~~

3. Behind a dataset, there is often an involved team with a wide range of skill levels. We must take into account their way of working, their work habits; so instead of wanting to change their habits completely, we must rather adapt them in a way that is beneficial to them, and make them actors in the data FAIRification process. Probably the best FAIR training is the one based on their data. But it is necessary to capitalize on good practices in written protocols, and referenced into data management plans.

4. Researchers must be involved in the data FAIRification process by providing them with tools for assessing their practices so that they can progressively improve them in stages, having properly integrated each of the criteria implemented. Especially, these assessment tools should highlight all the steps where small actions can significantly improve the FAIRification of the data. They will thus be more inclined to integrate them into their practice with full knowledge of the facts.

5. Concerning ~~Finally, a last word on~~ the data provenance: not only the authors, but above all the context, the methods of data acquisition and processing are crucial information for a good reuse. Unfortunately, this aspect is somewhat neglected, if not absent. An effort still needs to be made in this direction in order to sensitize and motivate data producers.

Finally, it should be mentioned that the license of the data must be appropriate for the reuse of the data.

Declarations

Ethics approval and consent to participate

Not applicable.

Consent for publication

Not applicable.

Availability of data and material

All information, documents, data and software concerning ODAM are accessible from Github [10]

Competing interests

The authors declare that they have no competing interests

Funding

DJ, RD and YG were partly supported by the PHENOME-EMPHASIS project funded by the French National Research Agency (ANR-11-INBS-0012). DJ and YG were also supported by the FRIMOUSS project funded by the French National Research Agency (ANR-15-CE20-0009-01). RD was also partly supported by the EPPN2020 project (H2020 grant N°731013), the EOSC-Life european program (grant agreement N°824087). SA was partly supported by the FooSIN project funded by the French National Research Agency (ANR- 19- DATA- 0019-01). All authors were also partly funded by the French National Research Institute for Agriculture, Food and the Environment (INRAE). The FRIM1 dataset came from research supported by the Eranet Erasysbio+ FRIM project funded by the French National Research Agency (ANR-09-SYSB-003) and the MetaboHUB project funded by the French National Research Agency (ANR-11-INBS-0010).

Author contributions

conceptualization: D.J; data curation: D.J; funding acquisition: Y.G; methodology: D.J, R.D; software: D.J; writing—original draft: D.J, R.D; writing—review and editing: D.J, R.D, S.A, Y.G. All authors read and approved the final manuscript.

Acknowledgements

We thank Catherine Deborde (PMB-Metabolome, INRAE, MetaboHUB) for advice on the manuscript and for constructive reviews.

References

1. Wilkinson MD, Dumontier M, Aalbersberg IJJ, Appleton G, Axton M, Baak A, et al. The FAIR Guiding Principles for scientific data management and stewardship. Sci Data. 2016;3:160018. doi:10.1038/sdata.2016.18.
2. European Commission Directorate General for Research and Innovation (2018) Turning FAIR into reality, Final Report and Action Plan from the European Commission Expert Group on FAIR Data, https://ec.europa.eu/info/publications/turning-fair-reality_en [Accessed June 04, 2020]
3. Jacobsen A, de Miranda Azevedo R, Juty N, Batista D, Coles S, Cornet R et al (2020) Data Intelligence 2: 1-2, 10-29 doi:10.1162/dint_r_00024
4. Bénard C, Biais B, Ballias P, Beauvoi B, Bernillon S, Cabasson C et al (2018), FRIM - Fruit Integrative Modelling, doi.org/10.15454/95JUTK, Portail Data INRAE, V3
5. Sansone, S., Rocca-Serra, P., Field, D. et al (2012), Toward interoperable bioscience data, Nat Genet 44, 121–126, doi:10.1038/ng.1054
5. ~~Data Package, Frictionless Data Specs https://specs.frictionlessdata.io/data-package/ [Accessed June 04, 2020]~~
6. Rocca-Serra P and Sansone SA (2019) Experiment design driven FAIRification of omics data matrices, an exemplar, Scientific Data volume 6, 271 doi:10.1038/s41597-019-0286-0

Formatted: No underline, Font color: Auto,

7. European Commission, Directorate-General for Research and Innovation (2018), Cost-Benefit analysis for FAIR research data - Cost of not having FAIR research data, https://op.europa.eu/en/publication-detail/-/publication/d375368c-1a0a-11e9-8d04-01aa75ed71a1 [Accessed June 04, 2020]

8. Wolstencroft K., Owen S., Krebs O., Nguyen Q. et all (2015) SEEK: A systems biology data and model management platform, BMC Systems Biology 9:33, doi:10.1186/s12918-015-0174-y

~~8.~~9. Leonelli S, Smirnoff N, Moor J (2013) Making open data work for plant scientists, Journal of Experimental Botany, pp. 4109–4117, doi:10.1093/jxb/ert273

~~9.~~10. David R, Mabile L, Specht A, Stryeck S, Thomsen M, Yahia M et al. (2020) FAIRness Literacy: the Achilles' Heel of applying FAIR Principles. Hal-02483307 https://hal.inrae.fr/hal-02483307

~~10. "ODAM: Deployment and User's Guide". GitHub 2020 https://inrae.github.io/ODAM/~~

Formatted

Figures

Figure 1: ODAM (Open Data for Access and Mining) is an Experiment Data Table Management System (EDTMS) based on good data management practices concerning data structuring and the description of structural metadata. Indeed, the strong point of the approach is to define metadata in depth, i.e. at the level of the data itself (i.e. metadata at column-level such as factors, variables...) and not only as a "hat" on the data set. Thus, having structural metadata allows datasets to achieve a higher level of interoperability and greatly facilitates functional interconnection and analysis in a broader context. (**A**) To simplify, we have considered here the first two tables of data from the experiment, namely the individuals (*plants.txt*) followed by the samples (*samples.txt*). The data must be well organized i.e. each variable forms a column, each observation forms a row, and each table is relative to an entity i.e. the same type of observational unit (plants, samples, ...), and a file must contain only one data table. Since all experimental data tables were generated in an experiment associated with a design of experiment, the data tables were acquired sequentially as the experiment progresses. A link must exist between each of them,

generally defined by identifiers. In our example, each sample is linked to the plant from which it comes from. (**B**) Furthermore, whatever the type of experiment, it requires a design of experiment involving individuals, samples or whatever, as the main objects of study and producing several tables of experimental data. It also involves the observation of dependent variables resulting from the effects of certain controlled independent variables (*factors*). In addition, the objects of study usually have an *identifier* for each one, and the variables can be *quantitative* or *qualitative*. Thus, each of the columns within a table (*attributes*) can be associated with one of the four categories: *identifier, factor, quantitative, qualitative.* By associating a category to each column, this greatly facilitate subsequent statistical analyses by the machines. All structural metadata can be grouped in two specific files. (**C**) The first metadata file associates to each data table (*subset*) a key concept corresponding to the main entity of the data table. It also defines for each table the link with the table from which it comes from (magenta arrows). These links can be interpreted as "is obtained from". (**D**) The second metadata file annotates each attribute (concept/variable) with minimal but relevant metadata, such as: its category defined above, its description with its unit, the data type. In each of these two files (entities and attributes), it is possible to annotate each of the terms with unambiguous definitions (CV terms) through links to accessible (standard) definitions based on ontologies. The choice of ontologies is very domain-specific but nevertheless it should preferably be based on those that follow the FAIR principles [r1]. In the case of the FRIM experiment, we mainly used AgroPortal [r2] and especially its "annotator" module made efficient thanks to the alignment of ontologies. Since these ontological terms are not essential for statistical analysis, they can be omitted up to the publication stage. It should be noted that tools for adding ontology terms to Excel spreadsheets are still being developed for ODAM software suite to facilitate this tedious task [r3]. Some tools such as RightField [r4], ISA-Tools [r5] or Swate [r6] offer interesting approaches and will be for sure good inspiration sources. Knowing that ontological terms are essential mainly for data dissemination, a connection with the ISA-TAB format for instance would make it possible to benefit from the tools already available for this type of task. In any case,

established mainly by and for the scientists who produced the data, this structural metadata will later allow non-expert users to explore and visualize the data, thus offering a better guarantee of correct (re)use by those who did not produce them. See Data Preparation Protocol for ODAM Compliance for more details (**Additional file 1**).

r1. A. Jacobsen et al (2020) Data Intelligence, doi:10-29 doi:10.1162/dint_r_00024
r2. C. Jonquet et al (2018) Computers and Electronics in Agriculture, doi:10.1016/j.compag.2017.10.012
r3. https://inrae.github.io/ODAM/todo last accessed: 2020-11-05
r4. https://rightfield.org.uk/ last accessed: 2020-10-15
r5. S. Sansone et al (2012) Nat Genet 44, 121–126, doi:10.1038/ng.1054
r6. https://github.com/nfdi4plants/Swate last accessed: 2020-10-15

Figure 1: ODAM-compliant structural metadata associated with the FRIM experimental data files: (**A**) Since all the experimental data tables were generated as part of an experiment associated with a Design of Experiment (DoE), each file thus contains data acquired sequentially as the experiment progressed. There must therefore be a link between each file, i.e. information that connects them together. In most cases, this information corresponds to identifiers. Thus, we can depict all data tables as a tree, the implicit link that connects them together being "obtained from". We need two specific files (**B &C**) to describe the structural metadata of the whole dataset, where only a part of each of them are shown here. (**B**) This metadata file makes it possible to associate each data subset with a key concept corresponding to the main entity of the subset, each subset being stored as a file (the grey rectangle). It also defines for each subset the link to the subset it originates (grey arrows). (**C**) This metadata file allows to annotate each attribute (concept/variable) with some minimal but relevant metadata, such as: its description with its unit, the data type, but also its category. (**D**) The category defined by controlled vocabulary (CV) is used to specify the type of each variable. In each of these two files (entities and attributes), it is possible to annotate each of the terms with unambiguous definitions through links to accessible (standard) definitions. Thus constructed, this metadata constitutes a dictionary describing each file (entity) as well as all the columns of the tables (attributes) offering a better guarantee in the correct (re)use of the data

Formatted: French (France)
Formatted: French (France)
Field Code Changed
Formatted: French (France)
Field Code Changed
Formatted: French (France)
Formatted: French (France)
Field Code Changed
Formatted: French (France)
Formatted: French (France)
Formatted: French (France)
Field Code Changed
Formatted: French (France)
Formatted: French (France)

~~for users who have not produced these data. See Data Preparation Protocol for ODAM Compliance for more details (doi:10.17504/protocols.io.betcjeiw).~~

Figure 2: ODAM software suite: In light blue (promote <-> provide) the engine of the approach, in purple the data and metadata provided by the user, in dark blue the activities related to the life cycle of the data. The whole process is implemented primarily to make better use of its data before its dissemination. The ODAM software embeds an API (Application Programming Interface) layer that allows interoperability between the different tables and the applications that will be able to use them. With the help of this layer, it opens up a whole ecosystem of potential applications, depending on your needs but also on your skills in the proposed tools. From the set of data files (which are non-combined tables, each corresponding to a particular observational unit that we name an entity), the user can: 1) Visualize the data associated with their metadata according to several criteria and in a completely interactive way with the help of the data explorer. 2) Export in tabular form subsets selected according to his criteria with combined, merged data. 3) Build and test his models more easily using a scripting language such as R, which allows it to repeat different scenarios according to a variety of parameters. All this is made possible thanks to the category as controlled vocabulary associated with each column, which facilitates statistical analysis by both humans and machines. Moreover, the first available data can be exploited as soon as the corresponding metadata have been captured without waiting until all the data are available.~~An R package allows to perform extractions according to the same criteria as those proposed in the data explorer.~~ The benefit of this approach is that the "life of the data" is integrated into the scientific research process, according to good data management practices that meet the essential FAIR criteria. Then, distributed data is enriched by associating a structural metadata file called datapackage [r1], a simple container which serves as metadata aggregator based on JSON schema specifications, an open, community-based interoperability standard. This compact and

Formatted: Font: (Default) Arial

Formatted: Font: (Default) Arial

hierarchically structured format proved to be suitable for integrating all of our structural metadata, thus placing the dataset in its experimental context, a key factor in making the data FAIR. Data generation according to this open schema is included in the proposed tools and does not require additional effort for the researcher. The definition of an explicit schema for structural metadata thus enables machines to better interpret the data for reuse. Indeed, exporting this metadata in datapackage format offers a great flexibility of use data via scripting languages such as R and Python on the basis of existing packages. Besides, this type of format allows a great variety in the choice of data repository as a distinct separation is established between structural metadata described in the datapackage format on the one hand, and descriptive metadata depending on the type of repository on the other hand. Preferably the chosen data repository should offer the ability to query and retrieve data using an API that conforms to the OpenAPI specification [r2] and that meet the essential criteria of the FAIR principles. For example, the following data repositories registered in re3data.org [r3] can be cited without being exhaustive: Dataverse [r4], Dryad [r5], FAIRDOMHub [r6], FigShare [r7], Zenodo [r8]. Finaly, t~~T~~he FAIRification can be considered from two points of view: 1/ It is linked to the data life cycle by the annotations and curations made on the data themselves, and to the quantity and quality of the information associated with the data (protocols, publications, keywords, ...), 2/ it can also be considered from the point of view of its data management practices, which must improve over time, which is precisely what the FAIR assessment grids attempt to measure, and more particularly the reproducibility and reusability of the data. See ODAM Deployment and User's Guide for more details [r9].

r1. https://frictionlessdata.io/ last accessed: 2020-10-15
r2. http://spec.openapis.org/oas/v3.0.3 last accessed: 2020-10-15
r3. http://re3data.org/ last accessed: 2020-10-15
r4. https://dataverse.org/ last accessed: 2020-10-15
r5. https://datadryad.org/ last accessed: 2020-10-15
r6. K. Wolstencroft et al (2017) Nucleic Acids Res, DOI : 10.1093/nar/gkw1032
r7. https://figshare.com/ last accessed: 2020-10-15
r8. https://zenodo.org/ last accessed: 2020-10-15
r9. https://inrae.github.io/ODAM/ last accessed: 2020-11-05

Formatted: Font: (Default) Arial

Formatted: French (France)

Formatted: French (France)

Formatted: French (France)

Field Code Changed

~~(https://inrae.github.io/ODAM/).~~

Figure 3: Interconnection of the different elements of the FRIM dataset from the Data INRAE repository [r1] as a hub (based on Dataverse ~~https://dataverse.org/~~). , a data repository that complies with the JSON-LD standard. Distributed data is enriched by associating a structural metadata file called datapackage [r2], a simple container format based on JSON schema specifications, an open, community-based interoperability standard. Schematically, the role of the data repository mainly ensures the "Findable" and "Accessible" criteria of the FAIR principles from the descriptive metadata, whereas the datapackage mainly ensures the "Interoperable" and "Reusable" criteria from the structural metadata, even if these roles are not exclusive. To be compliant with the FAIR principles, not all data, documents, workflows and other tools need to be located in a single system, but from a central repository, it is the set of links that constitutes the true information management system. It must be able to be traversed by a human being as well as by machines. By relying on explicit schemas (JSON-LD, JSON Schema) for both metadata and data, it becomes possible to reuse the data without friction, both by humans and machines. The use of spreadsheets greatly facilitates the annotation of data with metadata by the data producers themselves. Thus, this is technology, however powerful, that becomes part of the practices of non-experts in the domain, not the other way around. In addition, this further enhances the FAIR criteria, especially the reuse and interoperability criteria. To evaluate the level of the FAIRness, w~~W~~e used three FAIR grids, very different from each other. The first one, the OZONOME 5-star0 data ranking tool [r3], aims to perform an evaluation based on the FAIR principles as defined by Willkinson et al. [r4]~~(2016)~~. The main result is an overall rating, indicating the overall fairness of the data set. The other two grids are dedicated to a more refined assessment. The Fair Data Maturity Model (FDMM) document [r5] describes a maturity model for the FAIR assessment with indicators, priorities and assessment methods, which are useful for

Formatted: Font: (Default) Arial

Formatted: Font: (Default) Arial

Formatted: Font: (Default) Arial

Formatted: Font: (Default) Arial

Formatted: Font: (Default) Arial

Formatted: Font: (Default) Arial

Formatted: Font: (Default) Arial

Formatted: Font: (Default) Arial

Formatted: Font: (Default) Arial

Formatted: Font: (Default) Arial

Formatted: Font: (Default) Arial

Formatted: Font: (Default) Arial

Formatted: Font: (Default) Arial

standardizing assessment approaches in order to allow comparison of their results. Whereas the FAIR SHARC (SHAring Rewards and Credit) [r6] document allows the fairness of projects and associated human processes to be assessed, either by external evaluators or by the researchers themselves. Therefore, these grids cannot be compared with each other, but rather complement each other. Overall, the FAIRness of our dataset using the ODAM+Dataverse combination is of a good standard. However, to achieve complete FAIRification, we need to move towards semantic web approaches [r7]. By relying on explicit data schemas, the effort to climb this mountain can be envisaged with less fear.

Formatted: Font: (Default) Arial

Formatted: Font: (Default) Arial

r1. Institut National de Recherche pour l'Agriculture, l'Alimentation et l'Environnement. (2018). Data INRAE. DOI: 10.14758/9T8G-WJ20
r2. https://frictionlessdata.io/ last accessed: 2020-10-15
r3. https://confluence.csiro.au/display/OZNOME/Data+ratings last accessed: 2020-10-15
r4. MD. Willkinson et al (2016) Sci Data. DOI: 10.1038/sdata.2016.18
r5. https://www.rd-alliance.org/groups/fair-data-maturity-model-wg last accessed: 2020-10-15
r6. R. David et al (2020) Data Science. Journal. DOI: 10.5334/dsj-2020-032
r7. https://inrae.github.io/ODAM/todo last accessed: 2020-11-05

Formatted: French (France)

Formatted: French (France)

Field Code Changed

Formatted: English (United Kingdom)

Additional files

Additional file 1. Data Preparation Protocol for ODAM Compliance. The purpose of this protocol is to describe all the steps involved in collecting, preparing and annotating the data from an experiment associated with an experimental design (DoE) that will then allow the user to benefit from the services offered by ODAM.

Additional file ~~1~~2. FAIR evaluation of the FRIM1 dataset according to the 5 ★ Data Rating Tool grid. It aims to perform an evaluation based on the FAIR principles as defined by Willkinson et al. [1]. The main result is an overall rating, indicating the overall fairness of the data set.

Additional file ~~2~~3. FAIR assessment of the FRIM1 dataset according to the FDMM (FAIR Data Maturity Model) grid. This document describes a maturity model for the FAIR assessment with

indicators, priorities and assessment methods, which are useful for standardizing assessment approaches in order to allow comparison of their results.

Additional file ~~3~~4. FAIR assessment of the FRIM1 dataset according to the SHARC (Sharing Rewards and Credit) grid. This document allows the fairness of projects and associated human processes to be assessed, either by external evaluators or by the researchers themselves.

Figure 1

Click here to access/download;Figure;Figure1.png

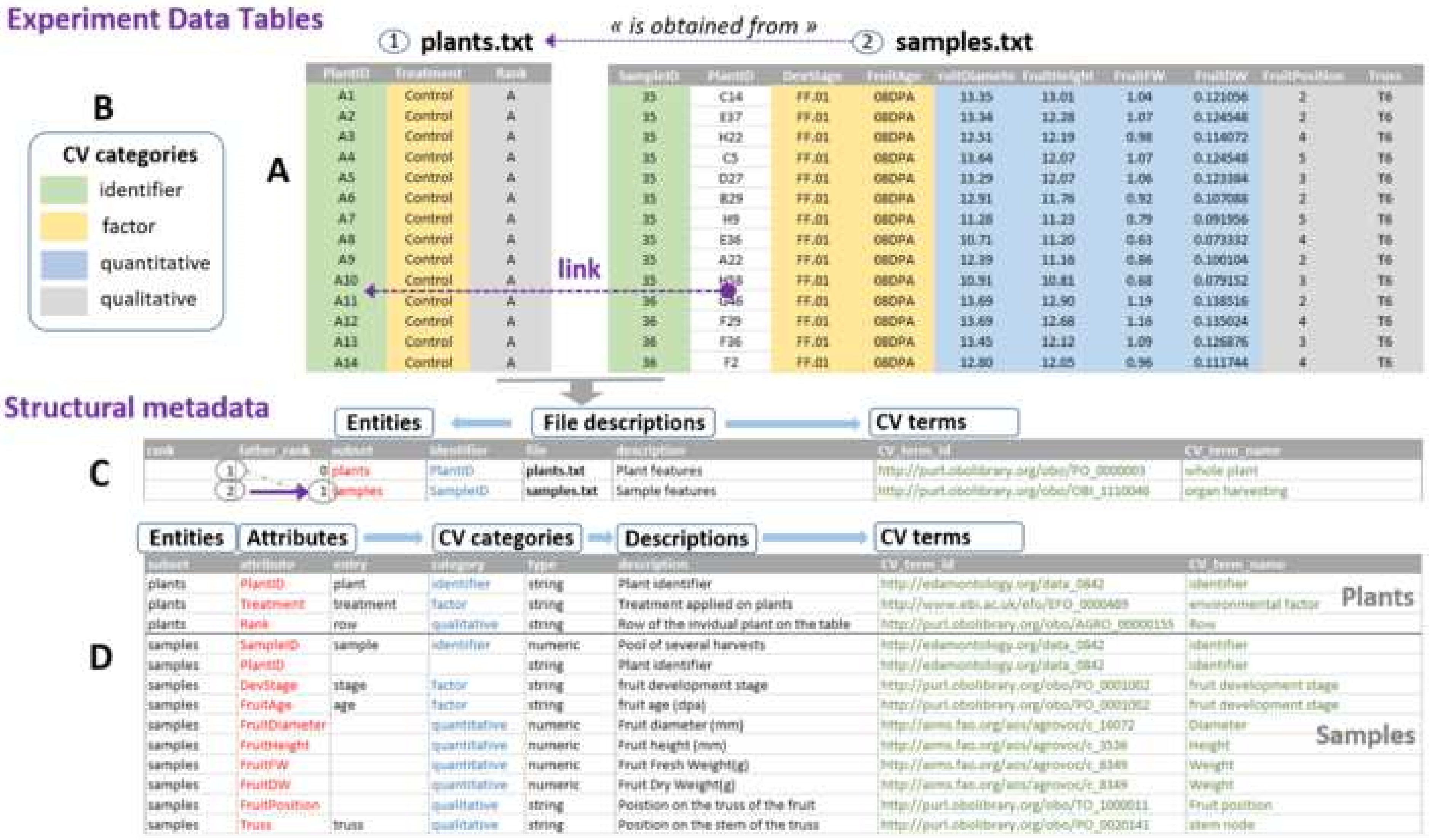

Figure 2

Click here to access/download;Figure;Figure2.png

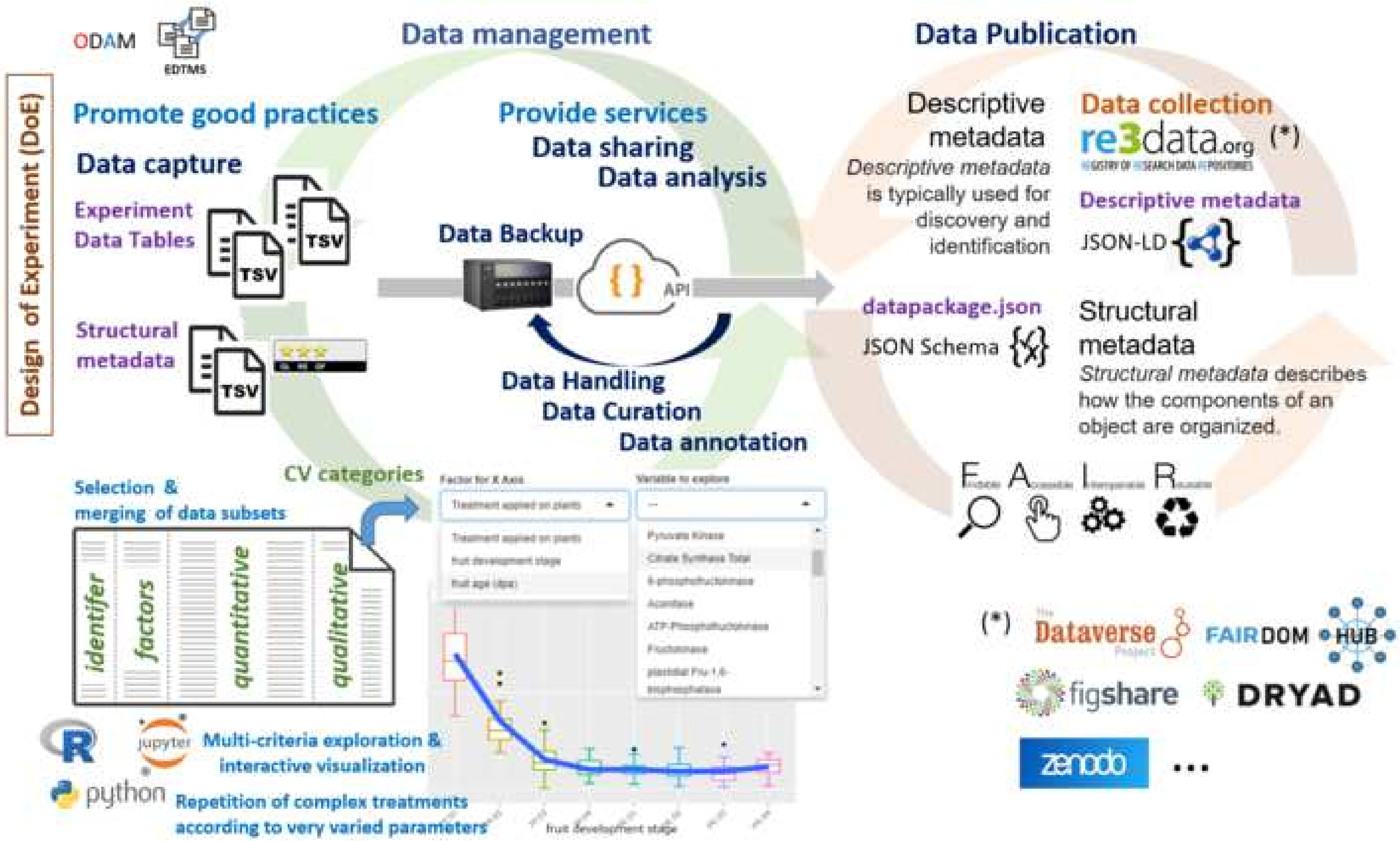

Figure 3

Click here to access/download;Figure;Figure3.png

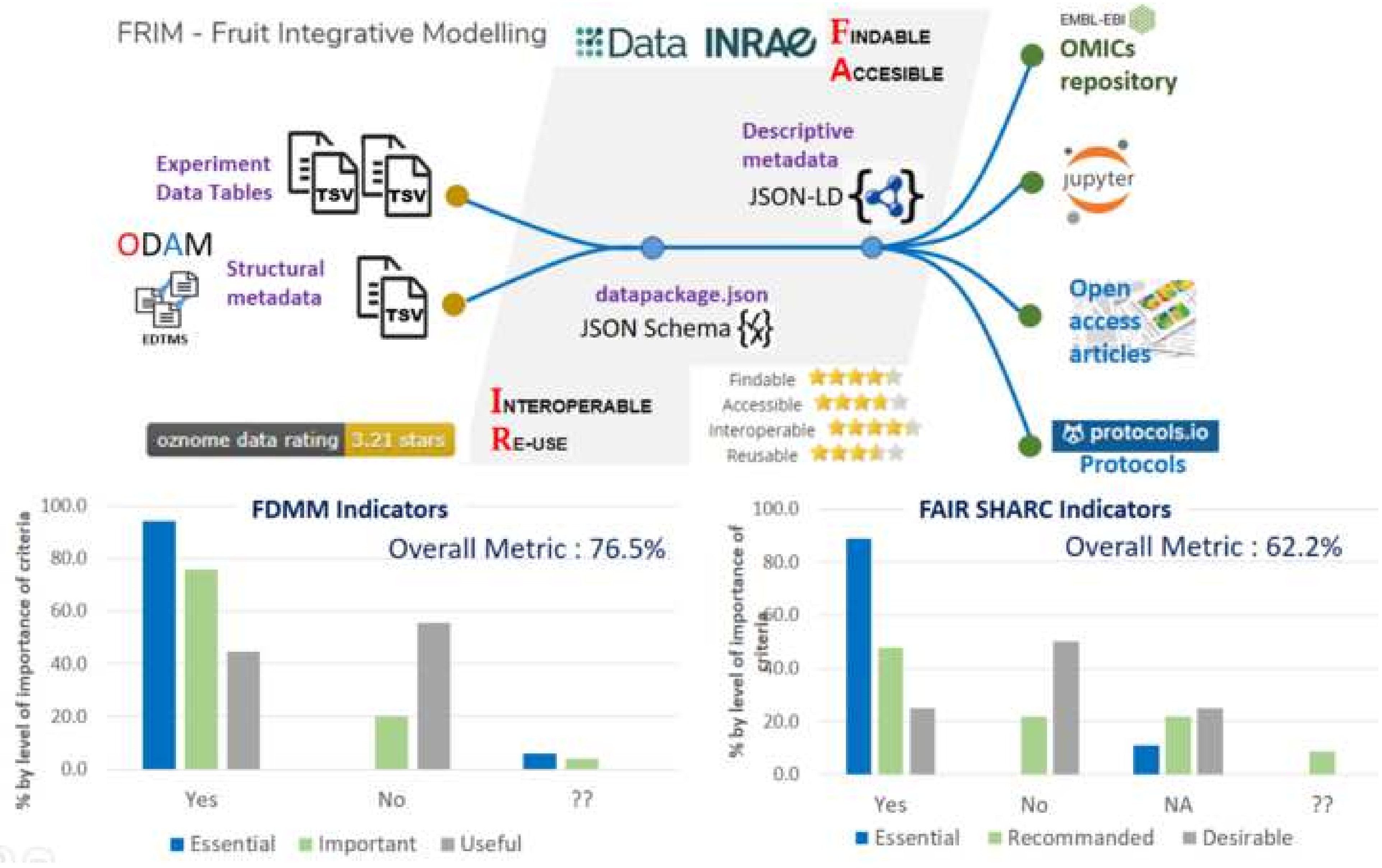

Additional file 1

Click here to access/download
Supplementary Material
Additional_file_01.pdf

Additional file 2

Click here to access/download
Supplementary Material
Additional_file_02.xlsx

Additional file 3

Click here to access/download
Supplementary Material
Additional_file_03.xlsx

Additional file 4

Click here to access/download
Supplementary Material
Additional_file_04.xlsx
