# Supplementary material for: Making experimental data tables in the life sciences more FAIR: a pragmatic approach
Source: Gigascience. 2020 Dec 15;9(12):giaa144. doi: 10.1093/gigascience/giaa144 (PMC7736789; doi:10.1093/gigascience/giaa144)
Supplement: giaa144_Supplemental_Files [file giaa144_supplemental_files.zip › Additional_file_01.pdf]

Data Preparation Protocol for ODAM Compliance

- Daniel Jacob[1]

- [1]INRAE Fruit Biology and Pathology, France

Abstract

ODAM (Open Data for Access and Mining) is an Experiment Data Table Management System (EDTMS) that implements a simple way to make research data broadly accessible and fully available for reuse, including by script languages such as R and Python. The main purpose is to make a dataset accessible online with minimal effort from the data provider, and to allow any data scientists or researchers to be able to explore the dataset and then extract a subpart or the totality of the data according to their needs.

The purpose of this protocol is to describe all the steps involved in collecting, preparing and annotating the data from an experiment associated with an experimental design (DoE) that will then allow the user to benefit from the services offered by ODAM. The overall approach is based on good data management practices concerning data structuring and the description of structural metadata.

Indeed, the strong point of the approach is to define metadata in depth, i.e. at the level of the data itself (i.e. metadata at column-level such as factors, variables...) and not only as a "hat" on the data set. Thus, having structural metadata allows datasets to achieve a higher level of interoperability and greatly facilitates functional interconnection and analysis in a broader context.

Keywords: Experiment Data Table Management System, Data, ODAM, Best practices

Description of the different stages of the protocol

Description of all the steps involved in collecting, preparing and annotating the data from an experiment associated with an experimental design (DoE). The overall approach is based on good data management practices concerning data structure and the description of corresponding structural metadata.

Based on an example:

- In order to illustrate the different stages of this protocol, we have chosen an example from an experiment on tomato fruits grown in a greenhouse. The aim of this study was to build a model of fruit growth. For this, a certain amount of data was required, and we will limit ourselves to some of them in order to simplify the size of the data set.
- See the complete example:

  Data explorer https://pmb-bordeaux.fr/dataexplorer/?ds=frim1
  Dataverse : https://doi.org/10.15454/95JUTK

1- Data Gathering

In our data subset example, we have 5 data files, one by type of object (plants, harvests, samples, compounds and enzymes).

- 5 different entities within the study, each corresponding to a file of data tables:
  plants, harvests, samples, compounds and enzymes
- 2 factors:
  Treatment, Development stages
- 53 quantitative variables:
  compounds (12) + enzymes (38) + weight, height, diameter (3)

1 - First, we put them under the same directory by giving it a name corresponding to the study or project (e.g. acronym of the project with a suffix corresponding to a study)

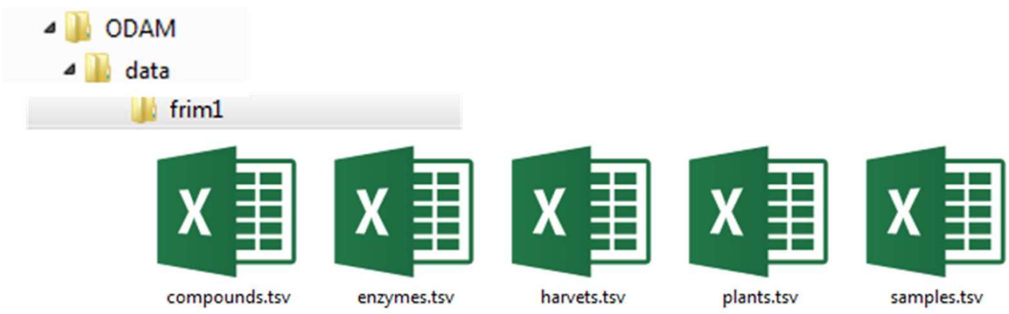

2 - Data subsets files must be compliant with the TSV standard (Tab-Separator-Values).

So an ODAM dataset is a bundle that contains a set of TSV files. The TSV files are simple tables containing the data of the dataset. In choosing this format, we follow the 5 gold stars principle, considered as a good practice and a necessary and indispensable step towards "Linked Open Data".

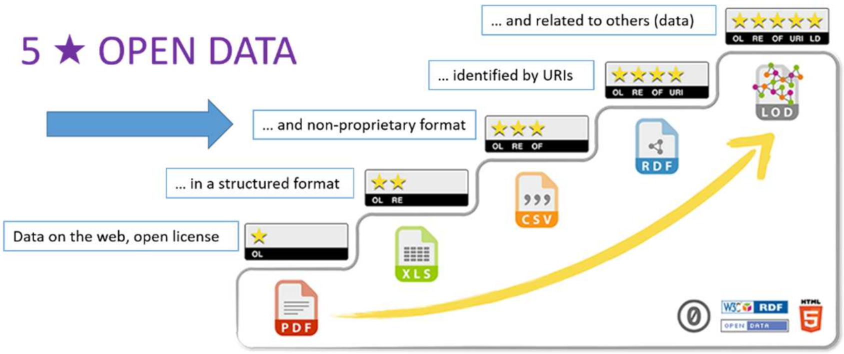

Tim Berners-Lee, the inventor of the Web and Linked Data initiator, suggested a 5-star deployment scheme for Open Data

Advice: To be sure to have the right format, do a 'copy' of data from the spreadsheet then 'paste' them into a new file, then 'save as TSV format (separator: a tab character)'

2 - Data structure and organization

Since all the experimental data tables were generated as part of an experiment associated with a Design of Experiment (DoE), each file thus contains data acquired sequentially as the experiment progressed.

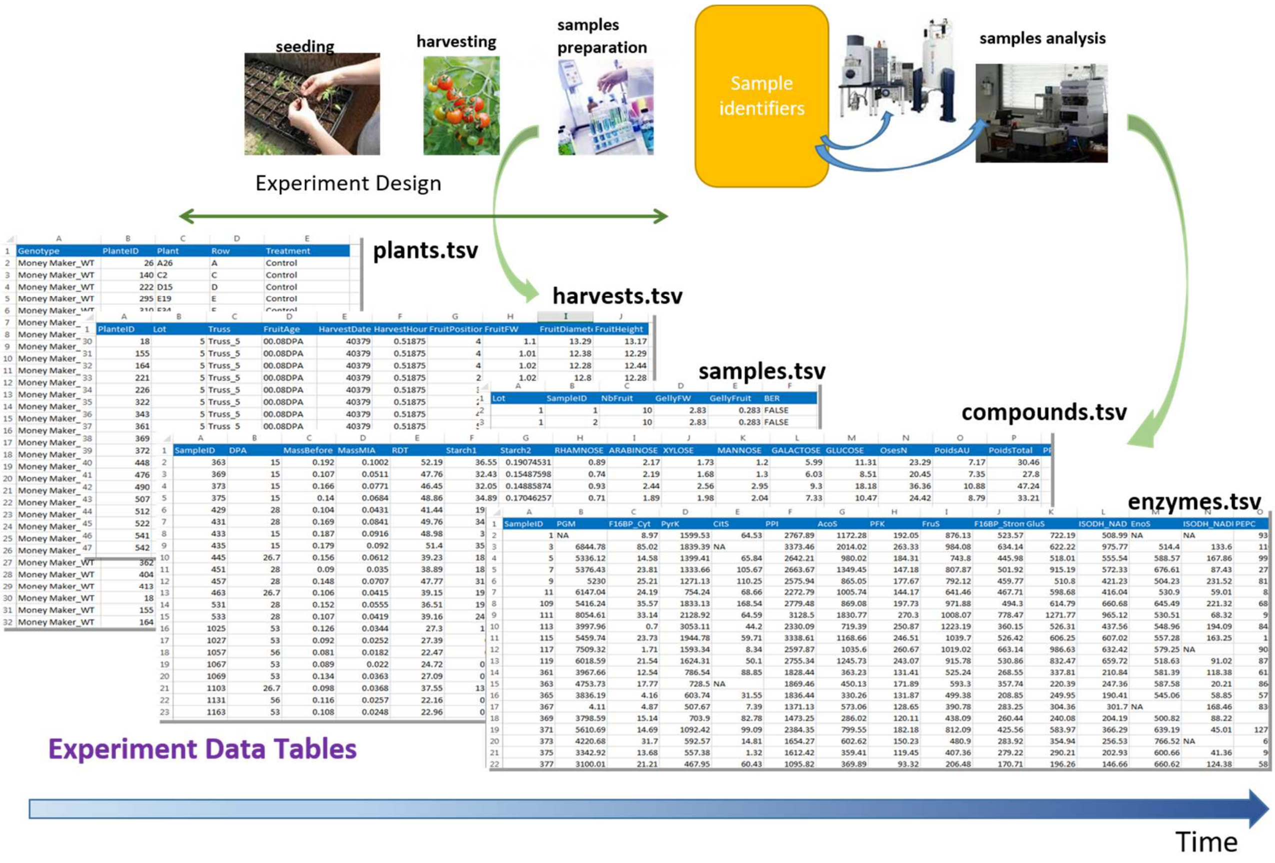

There must therefore be a link between each file, i.e. information that connects them together. In most cases (if not all), this information corresponds to **identifiers** that make it possible to precisely reference within the experiment each of the elements belonging to the same observation **entity** forming a coherent observation unit. For example, each plant, each sample has its own identifier, and each of these entities corresponds to a separate data file.

Well organized data means that each data table must be correctly structured, i.e.:

- Each variable forms a column,
- Each observation forms a line,
- Each type of "unit observational" (defined as an entity) forms a table, i.e. a file,
- Each data table file must have a column defined as an **identifier** (similar as a primary key) corresponding to each observation of the **entity** (e.g. plant, sample, …),
- Missing values can either be an empty cell or have the value 'NA',

- The header names must short without special characters. Only use the alphanumerical characters, and the underline character as word separator,
- The file should only contain data in matrix form and nothing else, i.e. no annotation on top, bottom, or sides.

Example of the ‘samples.tsv’ file:

samples : Sample features

A	B	C	D	E	F	G	H	I	J
SampleID	Treatment	DevStage	FruitAge	FruitPosition	FruitDiamete	FruitHeight	FruitFW	Rank	Truss
115	Control	FF.01	07DPA	3	11.95	10.42	0.81	A	T7
121	Control	FF.03	22DPA	3	36.13	31.77	21.43	A	T6
164	Control	FR.01	42DPA	2	51.09	46.85	64.05	A	T5
353	Control	FR.04	55DPA	5	48.28	43.35	66.64	A	T5
355	Control	FR.04	55DPA	3	49.84	44.93	66.98	A	T5
413	Control	FR.02	47DPA	1	60.48	54.23	106.13	A	T7
512	Control	FF.03	21DPA	NA	41	35.82	37.22	A	TA
117	Control	FF.01	07DPA	3	13.44	12.39	1.14	A	T7
536	Control	FR.02	47DPA	NA	59.4	49.05	87.28	A	TA
544	Control	FR.03	50DPA	NA	57.31	47.69	92.86	A	TA
158	Control	FF.04	35DPA	5	58.38	49.3	92.86	A	T5
109	Control	FF.03	22DPA	7	43.37	35.77	38.73	A	T5
134	Control	FF.02	15DPA	3	27.89	23.8	9.88	A	T7
31	Control	FF.01	08DPA	4	NA	NA	0.48	A	T6
179	Control	FF.03	28DPA	3	53.68	45.43	65.34	A	T7
383	Control	FF.04	34DPA	5	47.04	41.19	48.96	A	T7
425	Control	FR.04	55DPA	2	62.74	50.27	115.3	A	T7
520	Control	FF.03	30DPA	NA	48.86	41.52	52.94	A	TA
419	Control	FR.03	50DPA	2	55.63	48.02	86.79	A	T7
138	Control	FF.02	15DPA	6	27.96	22.14	9.69	A	T7
143	Control	FF.03	29DPA	4	48.45	42.92	51.35	A	T6
365	Control	FR.02	47DPA	5	55.11	44.9	71.82	A	T6
127	Control	FF.03	27DPA	3	45.71	43.28	47.8	A	T5
188	Control	FR.01	42DPA	3	55.38	47.1	77.39	A	T6

The files generated during data collection have to be organized according to the entity-relationship model similar to relational database management systems (RDBMS). Indeed, each **entity** corresponds to a type of collected data (samples, compounds, ...) for which is associated a set of **attributes**, i.e. a set of variables that may include observed or measured variables (quantitative or qualitative), controlled independent variables (factors) and an identifier.

Then, a link is established for each subset with the subset from which it was obtained, so that the links can be interpreted as "obtained from” as shown in the figure below:

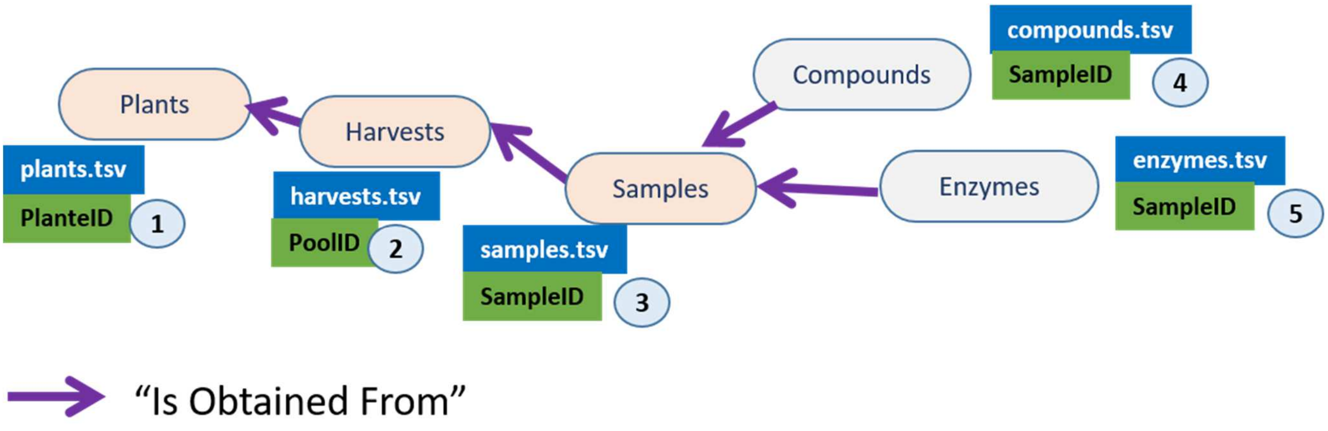

We have to organize your data subsets so that links could be established between them. In practical, it means to add when necessary a column (colored in green in the figure below) containing the identifiers corresponding to the entity to which we want to connect the subset. It is to be noted that this duplication of identifiers must be the only redundant information, through all data subsets.

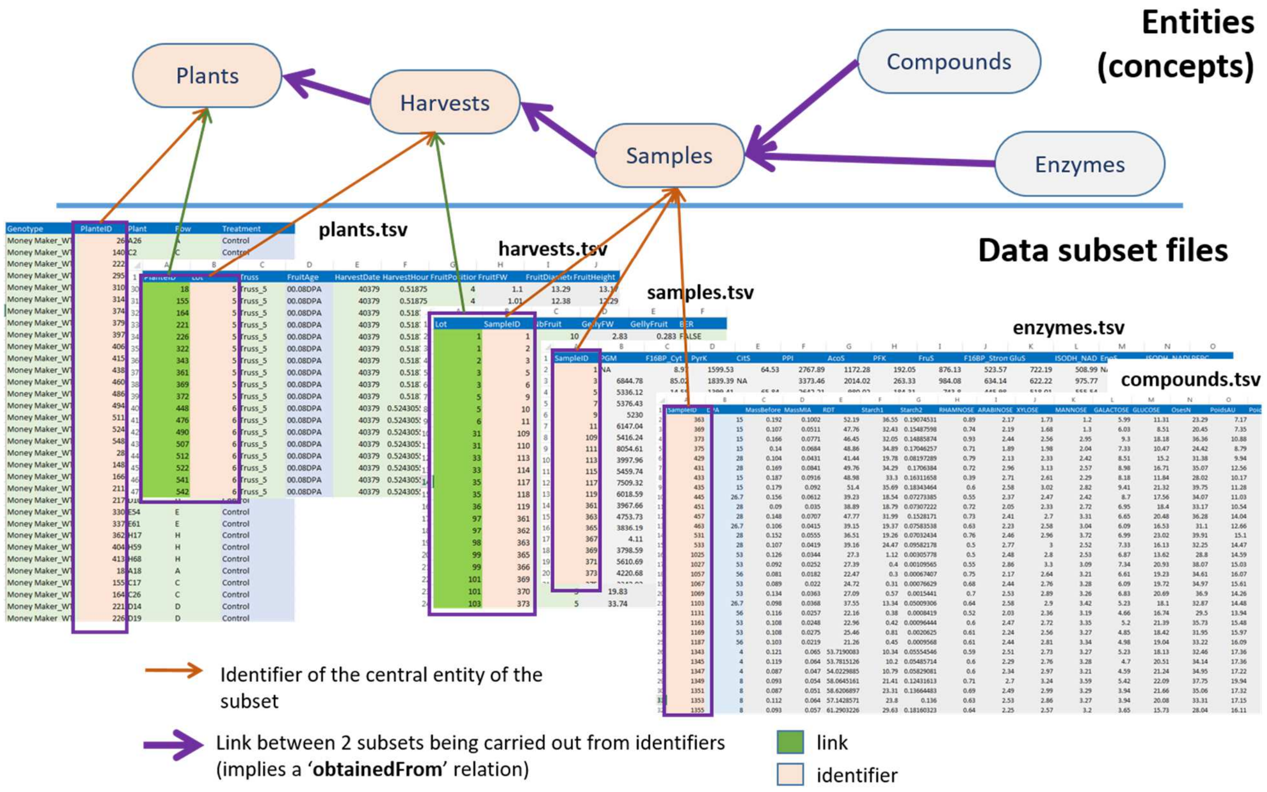

3 - Structural Metadata

Whatever the kind of experiment, this assumes a design of experiment (DoE) involving individuals, samples or whatever things, as the main objects of study (e.g. plants, tissues, bacteria, …). This also assumes the observation of dependent variables resulting of effects of some controlled experimental **factors**.
Moreover, the entities within the study have usually an **identifier** for each of them, and the variables can be **quantitative** or **qualitative**.

We can have either one entity within the study or several kinds, but in this latter case, it must exist <u>a relationship between entities that we assume of "obtained from" type</u> as describe above.

Thus, the data table of samples can be viewed according to the repartition by category we have just introduced as shown below:

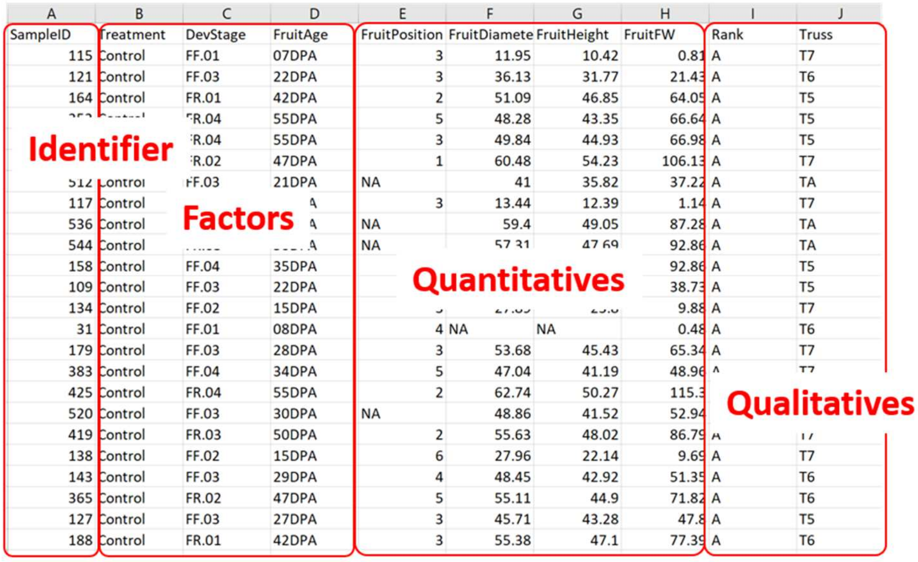

samples : Sample features

SampleID	Treatment	DevStage	FruitAge	FruitPosition	FruitDiamete	FruitHeight	FruitFW	Rank	Truss
115	Control	FF.01	07DPA	3	11.95	10.42	0.81	A	T7
121	Control	FF.03	22DPA	3	36.13	31.77	21.43	A	T6
164	Control	FR.01	42DPA	2	51.09	46.85	64.05	A	T5
[illegible]	[illegible]	[illegible]R.04	55DPA	5	48.28	43.35	66.64	A	T5
[illegible]	[illegible]	[illegible]R.04	55DPA	3	49.84	44.93	66.98	A	T5
[illegible]	[illegible]	[illegible]R.02	47DPA	1	60.48	54.23	106.13	A	T7
512	Control	FF.03	21DPA	NA	41	35.82	37.22	A	TA
117	Control	[illegible]	[illegible]	3	13.44	12.39	1.14	A	T7
536	Control	[illegible]	[illegible]	NA	59.4	49.05	87.28	A	TA
544	Control	[illegible]	[illegible]	NA	57.31	47.69	92.86	A	TA
158	Control	FF.04	35DPA	[illegible]	[illegible]	[illegible]	92.86	A	T5
109	Control	FF.03	22DPA	[illegible]	[illegible]	[illegible]	38.73	A	T5
134	Control	FF.02	15DPA	[illegible]	[illegible]	[illegible]	9.88	A	T7
31	Control	FF.01	08DPA	4	NA	NA	0.48	A	T6
179	Control	FF.03	28DPA	3	53.68	45.43	65.34	A	T7
383	Control	FF.04	34DPA	5	47.04	41.19	48.96	[illegible]	[illegible]
425	Control	FR.04	55DPA	2	62.74	50.27	115.3	[illegible]	[illegible]
520	Control	FF.03	30DPA	NA	48.86	41.52	52.94	[illegible]	[illegible]
419	Control	FR.03	50DPA	2	55.63	48.02	86.79	[illegible]	[illegible]
138	Control	FF.02	15DPA	6	27.96	22.14	9.69	A	T7
143	Control	FF.03	29DPA	4	48.45	42.92	51.35	A	T6
365	Control	FR.02	47DPA	5	55.11	44.9	71.82	A	T6
127	Control	FF.03	27DPA	3	45.71	43.28	47.8	A	T5
188	Control	FR.01	42DPA	3	55.38	47.1	77.39	A	T6

The four categories

- **Identifier**: precisely reference within the experiment each of the elements belonging to the same observation entity forming a coherent observation unit. For example, each plant, each sample has its own identifier,

- **Factor**: a factor of an experiment is a controlled independent variable; a variable whose levels are set by the experimenter. Treatments (control vs. stress), genotype (WT vs. mutant), the course of time (development stages) or even tissues, are typical factors of experiments.

- **Quantitative**: Quantitative data are values that describe a measurable quantity, in the form of numbers that can be calculated.

- **Qualitative**: Qualitative data describe qualities or characteristics. They answer questions such as "what type" or "what category". These values are no longer numbers, but a set of modalities. These values cannot be calculated.

In order to allow data to be ODAM-compliant, we have to adjoin some minimal but relevant metadata. For that, **two metadata files are required**.

Two specific TSV files, namely **s_subsets.tsv**and **a_attributes.tsv**, describe the metadata of the dataset, including informational metadata like descriptions of measures, as well as structural metadata like references between tables. The metadata lets non-expert users explore and visualize your data.

----

s_subsets.tsv

- a file allowing to associate with each subset of data a key concept corresponding to the main entity of the subset and the relations of the type "obtainedFrom" between these concepts

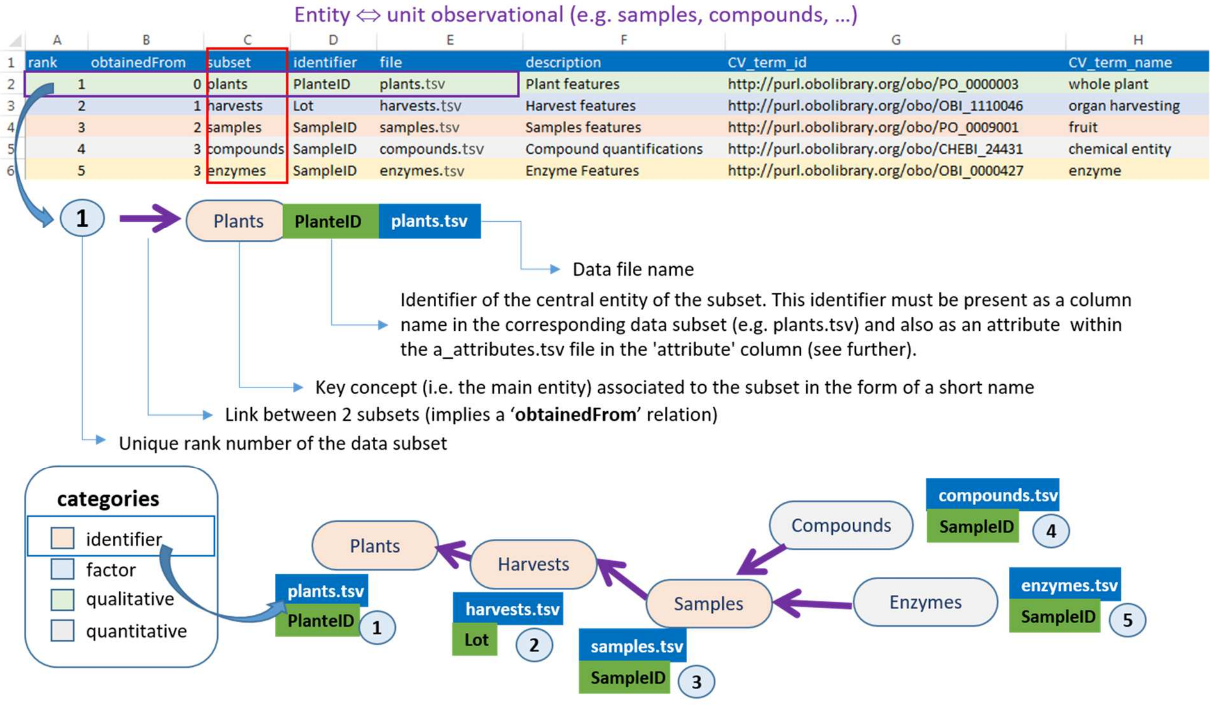

A column : Unique rank number of the data subset

B column : father rank

- the rank of the data subset (father rank) from which the data subset was obtained, implying a 'obtained from' relationship between both data subsets

C column : short name of the data subset

- i.e the entity name associated to the subset in the form of a short name
- only the alphanumerical characters and the underscore are allowed (i.e. 'a-z', 'A-Z', '0-9' and '_').

D column : The identifier attributes

- should be the only attribute declared as '***identifier***' in the '**category**' column in the **a_attributes.tsv**file (**D column**)
- should be available as a column item in the corresponding data subset file

E column : names of the files

- only the alphanumerical characters, the underscore and the dot are allowed (i.e. 'a-z', 'A-Z', '0-9' and '_' , '.')
- Moreover, these names should not start with a digit!

F column : description of the entity

- the allowed characters are: 0-9 a-z A-Z , : + * () [] {} - % ! | / . ?

G & H columns : annotations based on ontology

- use an ontology term (G) along with its corresponding URL (H)
- Sites such as [BioPortal]() or [AgroPortal]() are great sources for finding Control Vocabulary (CV) based on ontology
- These annotations are optional but it is mandatory to specify at least one so that the data table has exactly 8 columns. So a minimum good practice is to put for the first an NULL annotation e.g. 'NULL' (G), '/voc/null' (H).

----
a_attributes.tsv

- a metadata file allowing each attribute (concept/variable) to be annotated with some minimal but relevant metadata

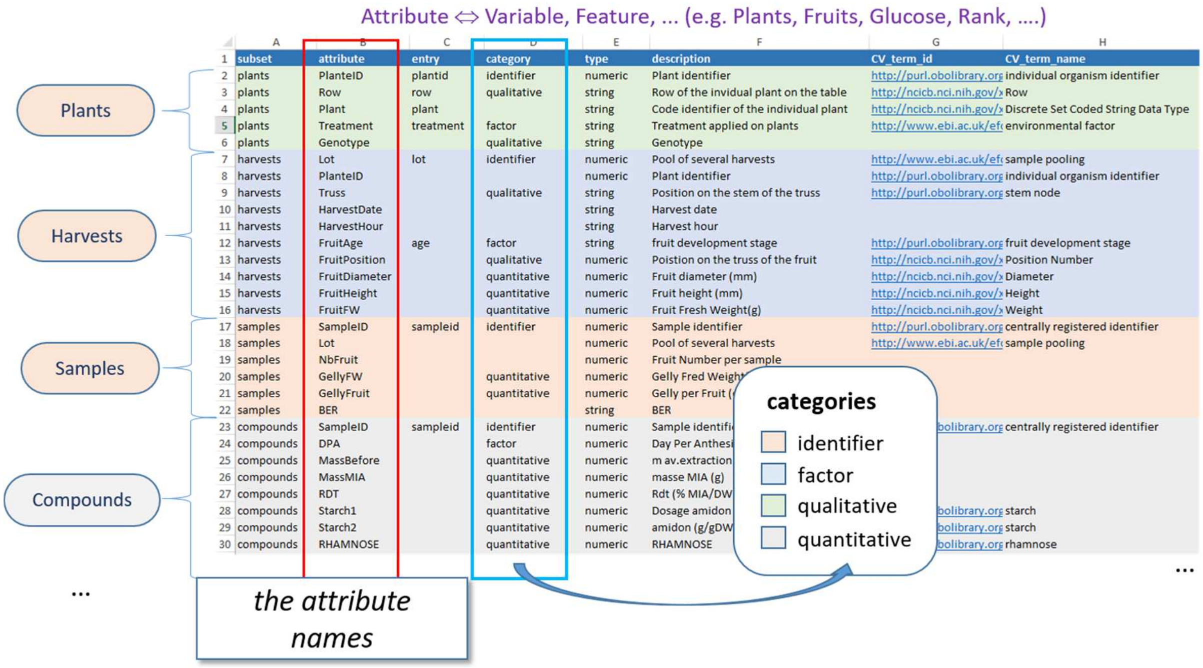

	A	B	C	D	E	F	G	H
1	subset	attribute	entry	category	type	description	CV_term_id	CV_term_name
2	plants	PlanteID	plantid	identifier	numeric	Plant identifier	http://purl.obolibrary.org	individual organism identifier
3	plants	Row	row	qualitative	string	Row of the invidual plant on the table	http://ncicb.nci.nih.gov/x	Row
4	plants	Plant	plant		string	Code identifier of the individual plant	http://ncicb.nci.nih.gov/x	Discrete Set Coded String Data Type
5	plants	Treatment	treatment	factor	string	Treatment applied on plants	http://www.ebi.ac.uk/efo	environmental factor
6	plants	Genotype		qualitative	string	Genotype		
7	harvests	Lot	lot	identifier	numeric	Pool of several harvests	http://www.ebi.ac.uk/efo	sample pooling
8	harvests	PlanteID			numeric	Plant identifier	http://purl.obolibrary.org	individual organism identifier
9	harvests	Truss		qualitative	string	Position on the stem of the truss	http://purl.obolibrary.org	stem node
10	harvests	HarvestDate			string	Harvest date		
11	harvests	HarvestHour			string	Harvest hour		
12	harvests	FruitAge	age	factor	string	fruit development stage	http://purl.obolibrary.org	fruit development stage
13	harvests	FruitPosition		qualitative	numeric	Poistion on the truss of the fruit	http://ncicb.nci.nih.gov/x	Position Number
14	harvests	FruitDiameter		quantitative	numeric	Fruit diameter (mm)	http://ncicb.nci.nih.gov/x	Diameter
15	harvests	FruitHeight		quantitative	numeric	Fruit height (mm)	http://ncicb.nci.nih.gov/x	Height
16	harvests	FruitFW		quantitative	numeric	Fruit Fresh Weight(g)	http://ncicb.nci.nih.gov/x	Weight
17	samples	SampleID	sampleid	identifier	numeric	Sample identifier	http://purl.obolibrary.org	centrally registered identifier
18	samples	Lot			numeric	Pool of several harvests	http://www.ebi.ac.uk/efo	sample pooling
19	samples	NbFruit			numeric	Fruit Number per sample		
20	samples	GellyFW		quantitative	numeric	Gelly Fred Weight [illegible]		
21	samples	GellyFruit		quantitative	numeric	Gelly per Fruit [illegible]		
22	samples	BER			string	BER		
23	compounds	SampleID	sampleid	identifier	numeric	Sample identifi[illegible]	[illegible]bolibrary.org	centrally registered identifier
24	compounds	DPA		factor	numeric	Day Per Anthesi[illegible]		
25	compounds	MassBefore		quantitative	numeric	m av.extraction [illegible]		
26	compounds	MassMIA		quantitative	numeric	masse MIA (g)		
27	compounds	RDT		quantitative	numeric	Rdt (% MIA/DW[illegible]		
28	compounds	Starch1		quantitative	numeric	Dosage amidon [illegible]	[illegible]bolibrary.org	starch
29	compounds	Starch2		quantitative	numeric	amidon (g/gDW[illegible]	[illegible]bolibrary.org	starch
30	compounds	RHAMNOSE		quantitative	numeric	RHAMNOSE	[illegible]bolibrary.org	rhamnose

A column : Short names of the data subsets

- must be declared in the **s_subsets.tsv** file (**C column**) and vice versa.

- only the alphanumerical characters and the underscore are allowed (i.e. 'a-z', 'A-Z', '0-9' and '_').

B column : attributes

- short name of the variables (data table column names)
- only the alphanumerical characters and the underscore are allowed (i.e. 'a-z', 'A-Z', '0-9' and '_').
- a set of variables that may include observed or measured variables (quantitative or qualitative), controlled independent variables (factors) and identifiers.
- one and only one attribute (**B column**) must be declared as '***identifier***' in the '**category**' column (**D column**) per data subset (**A column**)
- This column can be easily filled by copy-paste from the data table files, as shown below:

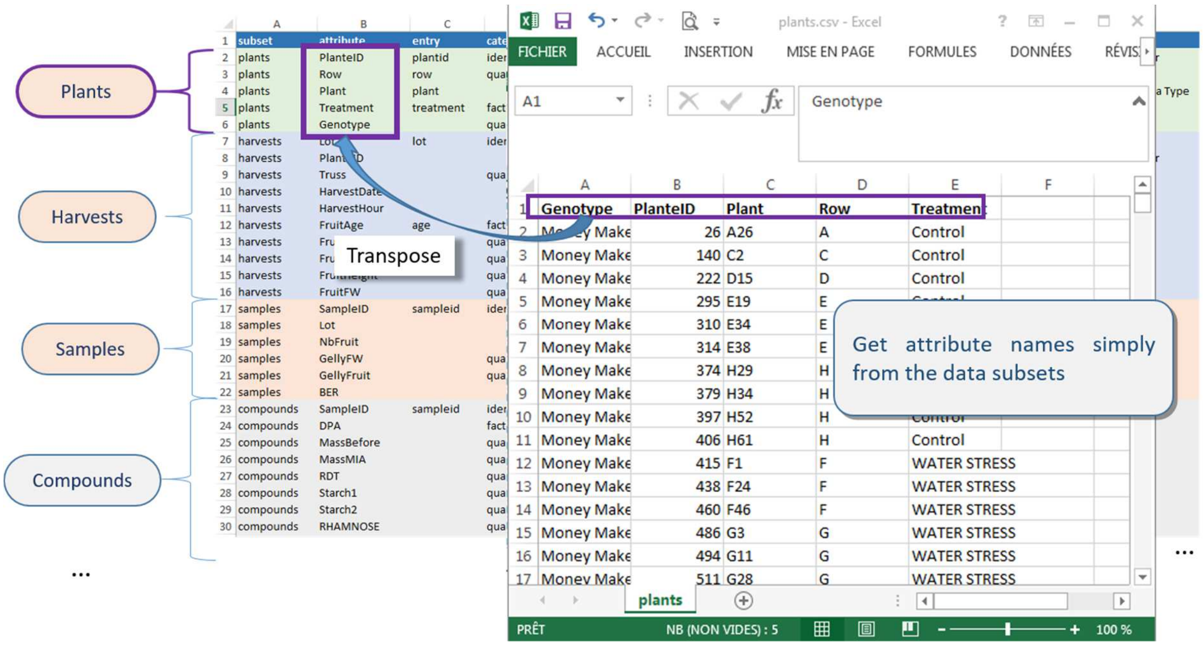

C column : Entry

- gives opportunity to make a selection on the attributes via web-services by associating them an alias name (called an “entry”).
- only the alphanumerical characters and the underscore are allowed (i.e. 'a-z', 'A-Z', '0-9' and '_').
- Example: we put ‘*treatment*’ as an entry for the ‘*Treatment*’ factor. By this, we could retrieve all samples data for Treatment equal to ‘*Control*’ by applying the API query: **http://myhost.org/getdata/tsv/frim1/(samples)/treatment/Control**
- See https://app.swaggerhub.com/apis-docs/INRA-PMB/ODAM/ for API testing

D column : Category

- has a limited choice of words: the set of terms are fixed, namely: '***identifier***', '***factor***', '***quantitative***', '***qualitative***'. Leave as blank otherwise.
- dependent variables resulting of effects of some controlled experimental factors must be defined as '***factor***'.

1. Each entity identifier must be defined as '***identifier***'
2. Variables can be defined as '***quantitative***' or '***qualitative***'.
3. External identifier which serves as a link must have an empty cell.

E column : data types

- the allowed names are restricted to '***numeric***' or '***string***'. All '*quantitative*' variables must be '*numeric*' type and it is preferable if the '*qualitative*' variables are '*string*' type.

F column : description of the attribute

- the allowed characters are: 0-9 a-z A-Z , : + * () [] {} - % ! | / . ?
- If a unit must be specified for a variable, we can add it in brackets at the end of the text of the description

G & H columns : annotations based on ontology

- use an ontology term (G) along with its corresponding URL (H)
- Sites such as BioPortal or AgroPortal are great sources for finding Control Vocabulary (CV) based on ontology
- These annotations are optional but it is mandatory to specify at least one so that the data table has exactly 8 columns. So a minimum good practice is to put for all attributes of type 'identifier' the corresponding annotation within the EDAM ontology , i.e. 'identifier' (G), http://edamontology.org/data_0842.(H)

Final checking

To complete this phase of data preparation, here is a list of some points to check and summarized below:

#	Note Description
**1**	A directory named as the dataset name should be actually created in the data repository;<br>Be careful in the spelling, see note 6;
**2**	The s_subsets.tsv and a_attributes.tsv files should be present in the data repository.
**3**	All data subset files declared in the s_subsets.tsv (col. E) should be available in the data repository
**4**	To be sure to have the right format, do a 'copy' of data from the spreadsheet then 'paste' them into a new file, then 'save as TSV format (separator: a tab character)'
**5**	1) all subsets in the a_attributes.tsv file (col. A) should be declared in the s_subsets.tsv file (col. C)<br>2) all subsets in the s_subsets.tsv file (col. C) should be declared in the a_attributes.tsv file (col. A)<br>3) all attribute names in the a_attributes.tsv file (col. B) should be available as a column in the corresponding data subset file declared in the s_subsets.tsv file (col. E)
**6**	Be careful in the spelling:<br>1) for data subset file names (col. E in s_subsets.tsv), identifier name (col. D in s_subsets.tsv), attribute names (col. B in a_attributes.tsv), subset short names (col. C in s_subsets.tsv and col. A in a_attributes.tsv) and entry names (col. C in a_attributes.tsv), only the alphanumerical characters and the underscore are allowed (i.e. 'a-z', 'A-Z', '0-9' and '_'). Moreover, these names should not start with a digit!<br>2) for categorical names (col. D in a_attributes.tsv), the number of terms and their spelling are fixed, namely: 'identifier', 'factor', 'quantitative', 'qualitative'.<br>3) for type (col. E in a_attributes.tsv), the allowed names are restricted to 'numeric' or 'string'.<br>4) for description, the allowed characters are: 0-9 a-z A-Z , : + * () [] {} - % ! | / . ?
**7**	Identifiers declared in the s_subsets.tsv file (col. D)<br>1) should be declared as 'identifier' in the 'category' column in the a_attributes.tsv file (col. D)<br>2) should be available as a column item in the corresponding data subset file<br>3) should be the only one attribute declared as identifier for the corresponding data subset file in the a_attributes.tsv file (col. D)
**8**	Each subset having a 'father_rank' greater than 0 in the s_subsets.tsv file (col. B)<br>1) should include in its data file a column corresponding to the identifier of the subset to which it is linked (i.e. corresponding to the father_rank in col. A)<br>2) should have the linked subset identifier with no category (i.e. empty) in the a_attributes.tsv file (col. D), except if the subset and the linked subset have the same identifier

Fortunately, all of these checks can be done for you.

- See how it looks for our complete online example:
  - https://pmb-bordeaux.fr/getdata/check/frim1

How to install and configure ODAM.

- See https://inrae.github.io/ODAM/

This document is also available online with a DOI

Daniel Jacob. Data Preparation Protocol for ODAM Compliance. protocols.io
https://dx.doi.org/10.17504/protocols.io.betcjeiw

LICENSE

This is an open access document distributed under the terms of the Creative Commons Attribution License, which permits unrestricted use, distribution, and reproduction in any medium, provided the original author and source are credited.
