# Supplementary material for: Making experimental data tables in the life sciences more FAIR: a pragmatic approach
Source: Gigascience. 2020 Dec 15;9(12):giaa144. doi: 10.1093/gigascience/giaa144 (PMC7736789; doi:10.1093/gigascience/giaa144)
Supplement: giaa144_Response_to_Reviewer_Comments_Original_Submission [file giaa144_response_to_reviewer_comments_original_submission.pdf]

Author's Response To Reviewer Comments

Close

Dear Editor,

Concerning manuscript "Towards FAIRification of experimental data tables in life sciences: a pragmatic approach" (GIGA-D-20-00198), all remarks of reviewers 1 and 3 have been taken into account. A response was also made to reviewer 2 for whom we think his judgment seems a bit hasty but we ve taken in account his remarks as much as possible by rewording.
Reviewers 1 and 3, experts in the field, provided us with valuable revision elements to improve the manuscript. We were keen to satisfy the various advice and remarks, especially since they were provided in a spirit of benevolence with a real interest for the content of the manuscript. The type of paper being a commentary, i.e. a short paper, we wanted to keep the initial structure of the manuscript, i.e. a more general argument in the main text, and more technical details in the figures and their captions. Thus, considering the limit imposed (1500 words, 10 references), we nevertheless tried to answer as much as possible to the whole advices and suggestions.
The answers and changes that have been made are detailed below.

Please let us know if some additional modifications are needed.

Best wishes,

Daniel Jacob

Reviewer reports:
=============

Reviewer #1:

Dear Authors,

I enjoyed your manuscript very much, as it addresses the difficult realities of trying to bring FAIRness to the source of the data! As the institutional data steward for my institute, I resonated very much with the issues you discussed in this submission.

Answer: We are very pleased with your interest in our work.

I have a few comments that might improve the submission in several ways.

1) I don't think you have given sufficient acknowledgement of prior art, especially around the integration of semantic data capture into spreadsheets. Acknowledging (at least) the Right Field project and the ISA-toolkit would be appropriate (and maybe even discussing if Right Field/ISA-Tools can work in-parallel with the structured approach you refer-to in the protocols.io submission, Figure 1 legend)

Answer: Indeed, although being an important point we neglected it a bit in the first version. Partly because this point is still under development regarding our approach (cf https://inrae.github.io/ODAM/todo/). We have mentioned this point at the end of the Figure 1 legend by citing the suggested tools plus another very promising one as well.

2) Regarding the protocols.io submission that you refer-to in the Figure 1 legend - this is a very significant contribution, in my opinion! I feel it is sufficiently important that it should probably be discussed more directly, and in more detail, in this article. I finished reading the article not fully understanding what you meant, but after reading your Data Preparation Protocol for ODAM Compliance

submission to protocols.io, I had a much better understanding (and I might try to duplicate that approach in my own institute!). Unfortunately, I don't think that Figure 1 adequately explains what you are doing/proposing, and so it was a bit disappointing that I had to go to that reference to fully understand your paper.

Answer: It is true that without the associated protocol file, it was difficult to fully understand the process. Therefore, we have redone Figure 1 and completely rewritten its legend, in particular by taking up again important points mentioned in the protocol. Moreover, we have partially rewritten the " To promote good practices, provide services " section to better clarify the purpose of our work.
In addition, we have associated the protocol file as an additional file to the article so that it is now an integral part. In this way, we hope to have made our approach more readable.

3) Another piece of software that I think would be appropriate to mention is the SEEK data management platform, from the FAIR-DOM project. Project-level and dataset-level metadata and provenance capture is handled by this open-source tool quite nicely!

Answer: The strength of our approach according to us, being the structural metadata associated with the data, we wanted to focus our paper more on this point. Thus we distinguish between descriptive metadata, i.e. the overall context on the one hand and structural metadata, i.e. metadata describing the interconnections between the data along with the functional categories on the other hand. The data and their metadata formatted according to our approach allows users to have a great flexibility in the choice of data repository. We have given some examples of this in Figure 2. Regarding the SEEK data management platform, we actually found it very relevant indeed, so we have mentioned it in the main text.

4) Numerous grammatical errors throughout. I captured some of them, but there are others that i didn't highlight:

line 29 remove "a" from "a research"
Answer: Ok, corrected.

line 57: remove "many"
Answer: Ok, corrected

line 68/69: pronoun missing "allowing to":
Answer: As the text has changed, this is no longer applicable. But indeed, we have paid attention to the use of these verbs in English (allow X to, enable X to) in this paper. Thank you.

line 75/76: bad grammar: because essentially using a spreadsheet
Answer: Ok, corrected

line 82/83: I don't understand this sentence: Better still, it opens up data on a whole ecosystem of potential applications, according to their needs and skills
Answer: We have rewritten this sentence as this: "Besides, depending on the needs and skills of researchers, data can be used in a wide variety of ways". See lines 87-88

lines 86-88: the sentences that span those lines needs to be grammatically corrected.
Answer: We have rewritten this sentence as this: "In doing so, FAIRification of data is carried out in order to handle data more efficiently and not just to publish it". See lines 91-92

line 91: pronoun missing "enables to"
Answer: Corrected

line 122: the second item in this list is referring (I guess?) to "researchers", the first item in the list. This is not how the list is introduced (Summary of the proposed approach and beyond). that list item needs to be restructured to match the form of the other list items.
Answer: Ok we have rewritten this sentence. See lines 130-135

Thank you for your efforts in writing this manuscript! I wish you luck on publication!
Answer: Thank you for your encouragement, very appreciated!

Sincerely,

Mark Wilkinson

Reviewer #2:

The authors provide a short summary of ways to make data publication compliant with FAIR principles (findable, accessible, interoperable, reusable). These principles are universally desirable and intuitive -- they describe requirements for sound research and communication of findings.

The paper describes ways of fixing the problem of spreadsheets without necessarily taking the spreadsheets away from the researchers. I would not agree that they are a "tool par excellence". They are convenient -- available and with a low bar for entry. Ironically, it is this very convenience that makes them unreliable for storage and analysis.

The paper hinges on 5 points, listed in the "Summary of proposed approach". My interpretation of these points is the following

1. Make data available through a service that is tailored to the needs of the researcher and their expertise in data wrangling.
2. Create a database schema, normalize the data and map it onto fixed vocabulary or ontology.
3. Train researchers to adopt FAIR principles a la carte and where it is both practical and useful for them to do so. Thus, as opposed to transitioning to a fully "FAIRized" system, stagger the adoption and training process and use the researcher's own data for training. Essentially, use a bit of social engineering to get people to do what you feel is good for them.
4. Have a way to assess whether implementing new data handling methods is adding benefit.
5. Every aspect of data collection, handling, analysis and publication should be designed with FAIR principles in mind.

These 5 points are true but, in my mind, they are so true that they are essentially widely accepted as necessary. For example, a fixed vocabulary and normalized database schema for data storage and lookup are common solutions to the problem of consistency and querying. I don't think that points like this need to be emphasized anymore.

The workflow shown in Figure 2 is flexible and sophisticated but illustrates steps that are required in any research venture that is sound (e.g. data backup, metadata, visualization).

Answer:

We agree overall with your analysis, except that:
The FAIR principles are widely accepted, but this does not mean that they are easily adopted by all for data dissemination (e.g. in re3data.org more than 50% of the repositories do not have a Persistent Identifier!). There is still a long way to go (way of the cross?) before we get there.
Beyond the principles, it is above all the practical aspects that make it possible to get there. It is on these aspects that our paper focuses, especially for experimental data tables. For this type of data in particular, interoperability criteria require structural metadata for fostering reuse. However, regardless of the skills required to build a data model, it seems unreasonable to us to have to build such models in order to constitute a database each time a greenhouse or field experiment is carried out. This approach is simply out of touch with the realities in most cases. This is why, in such cases, we need to be very pragmatic in proposing approaches that are easy to implement within a reasonable timeframe, and that do not necessarily require strong IT engineering skills. This is what we try to propose in this paper concerning more specifically experimental data tables.

We agree with you that "a tool par excellence" was a bit excessive. So we changed that to "a tool that researchers master very well".

Reviewer #3:

This well written paper aims to describe how researchers can prepare their data to be more FAIR, taking the specific example of plant data. The paper lays out a set of pragmatic guidelines to move the community closer to FAIRer data and good data management in general and concludes with an assessment of their test data via a number of recent FAIR data assessment frameworks.

There are many good pieces of advice in the paper and all reasoning is well explained. However, the focus on spreadsheet data, while understood, given spreadsheets are, in the words of the paper, 'the tool par excellence', does limit the scope somewhat, as some of the guidelines could be expanded to other types of data. However, given this focus, the paper would benefit from having more examples, explanations and recommendations of specific controlled vocabularies or reporting guidelines to follow (e.g. Why was ODAM chosen? Which CVs were chosen, are the CVs FAIR?)

Answer: In this paper, we wanted to focus on the benefit of FAIRification integrated into the data management process based on good practices, and not on FAIRification in general. In addition, as the title indicates, we have illustrated it from tables of experimental data by proposing a specific approach to this type of data. This document being a commentary, i.e. a short paper (1500 words maximum, 10 references maximum) did not allow us to develop all the points of FAIRification.
Concerning FAIRification in general and more specifically on the respect of the FAIR principles, we rely on a very recent paper (Jacobsen et al 2020, given in the references) which gives all the necessary information.

Nevertheless, concerning the points that you mention, we have added elements or precision allowing us to answer them in part because of the limit imposed on the size of the text, as follow:
- Why was ODAM chosen? see lines 67-75, core text.
- Which CV were chosen? are the CVs FAIR? see lines 243-246, figure 1 legend

I have two further specific points:

1. The authors choose (on line 90) the Frictionless Data JSON specifications as their interoperability standard of choice. This choice should be explained as many other interoperability standards are also available. This explanation could also touch on the use of metadata to place the dataset into context, a key factor to make the data FAIR, which is mentioned in the Frictionless Data specification but not in the main text.

Answer: Indeed, we had somewhat omitted this aspect in the first version of our manuscript. This is why we have remedied it in the figure 2 legend, lines 283-290. In addition, we have explicitly mentioned the specification in the main text. See line 97.

2. One issue that hasn't been covered in the Publication of data section (line 98-115) is that of data licensing. This is an important issue in FAIR and should, in my opinion, be mentioned in this section.

Answer: This is one of the aspects that would require further development but would be too much for this comment. Nevertheless, we have briefly mentioned this important point. See lines 151-153

For clarity, I would suggest the following additions to the text:

1. Line 90 - state the open, interoperability standard, rather than just the reference (so readers don't have to go to the reference list to find the name of the standard).

Answer: Right! We have mentioned the name of the standard in the main text. See line 97.

2. In general, the standards mentioned in the figures should also be mentioned in the text, as the figures seem slightly disconnected at present. For example, the use of ODAM in figure 1. ODAM isn't mentioned in the main text but is integral to the work.

Answer: Right! We have mentioned ODAM in the main text. See line 73.

There are a few typographical errors:

1. line 39 'e.g. *the* European commission'
Answer: Ok, corrected

2. line 59-60 'Thus, a tool *enabling the automatic combination of* data sets'
Answer: As the text has changed, this is no longer applicable. But indeed, we have paid attention to the use of these verbs in English (allow X to, enable X to) in this paper. Thank you.

3. line 75 'because *they are* essentially using'
Answer: As the text has changed, this is no longer applicable.

4. line 122 '2. *Researchers have* the best control'
Answer: Ok, corrected

The paper may also benefit from the addition of a reference on line 103 - 'Journal editors and reviewers are increasingly recommending that all data, complete and non-synthetic, generated during the course of the study be made available' - it would be good to provide a reference as evidence of this (I don't doubt the veracity, I just think it should be accompanied by a reference).

Answer: We looked for a reference that could support this opinion (based on our own observations when submitting papers), but found nothing satisfactory. Ideally, it could have been a survey of publishers and reviewers. The only one we found does not directly concern this point (see below). So we decided to delete this sentence, which saved us a few extra words (valuable in a short paper!).

Survey on open peer review: Attitudes and experience amongst editors, authors and reviewers
https://journals.plos.org/plosone/article?id=10.1371/journal.pone.0189311

I'd like to conclude by congratulating the authors. I particularly enjoyed and agree with the statement that starts on line 94 - 'So, in our approach data FAIRification is closely related to data management, avoiding a retroactive process that would require more time, costs and computer skills'.

This paper has many merits, not least as an example of how FAIR can and should be put into practice.

Answer: Thank you for your encouragement, very appreciated!

Close
