# Supplementary material for: Making experimental data tables in the life sciences more FAIR: a pragmatic approach
Source: Gigascience. 2020 Dec 15;9(12):giaa144. doi: 10.1093/gigascience/giaa144 (PMC7736789; doi:10.1093/gigascience/giaa144)
Supplement: giaa144_Reviewer_1_Report_Original_Submission — Mark Wilkinson -- 7/14/2020 Reviewed [file giaa144_reviewer_1_report_original_submission.pdf]

Reviewer Report

Title: Towards FAIRification of experimental data tables in life sciences: a pragmatic approach

Version: Original Submission **Date:** 7/14/2020

Reviewer name: Mark Wilkinson

Reviewer Comments to Author:

Dear Authors,
I enjoyed your manuscript very much, as it addresses the difficult realities of trying to bring FAIRness to the source of the data! As the instiututional data steward for my institute, I resonated very much with the issues you discussed in this submission.
I have a few comments that might improve the submission in several ways.
1) I don't think you have given sufficient acknowledgement of prior art, especially around the integration of semantic data capture into spreadsheets. Acknowledging (at least) the Right Field project and the ISA-toolkit would be appropriate (and maybe even discussing if Right Field/ISA-Tools can work in-parallel with the structured approach you refer-to in the protocols.io submission, Figure 1 legend)
2) Regarding the protocols.io submission that you refer-to in the Figure 1 legfend - this is a very significant contribution, in my opinion! I feel it is sufficiently important that it should probably be discussed more directly, and in more detail, in this article. I finished reading the article not fully understanding what you meant, but after reading your Data Preparation Protocol for ODAM Compliance submission to protocols.io, I had a much better understanding (and I might try to duplicate that approach in my own institute!). Unfortunately, I don't think that Figure 1 adequately explains what you are doing/proposing, and so it was a bit disappointing that I had to go to that reference to fully understand your paper.
3) Another piece of software that I think would be appropriate to mention is the SEEK data management platform, from the FAIR-DOM project. Project-level and dataset-level metadata and provenance capture is handled by this open-source tool quite nicely!
4) Numerous grammatical errors throughout. I captured some of them, but there are others that i didn't highlight:
line 29 remove "a" from "a research"
line 57: remove "many"
line 68/69: pronoun missing "allowing to"
line 75/76: bad grammar: because essentially using a spreadsheet
line 82/83: I don't understand this sentence: Better still, it opens up data on a whole
83 ecosystem of potential applications, according to their needs and skills
lines 86-88: the sentences that span those lines needs to be grammatically corrected.
line 91: pronoun missing "enables to"
line 122: the second item in this list is referring (I guess?) to "researchers", the first item in the list. This is not how the list is introduced (Summary of the proposed approach and beyond). that list item needs to be restructured to match the form of the other list items.

Thank you for your efforts in writing this manscript! I wish you luck on publication!
Sincerely,
Mark Wilkinson

Level of Interest

Please indicate how interesting you found the manuscript: Choose an item.

Quality of Written English

Please indicate the quality of language in the manuscript: Choose an item.

Declaration of Competing Interests

Please complete a declaration of competing interests, considering the following questions:

- Have you in the past five years received reimbursements, fees, funding, or salary from an organisation that may in any way gain or lose financially from the publication of this manuscript, either now or in the future?
- Do you hold any stocks or shares in an organisation that may in any way gain or lose financially from the publication of this manuscript, either now or in the future?
- Do you hold or are you currently applying for any patents relating to the content of the manuscript?
- Have you received reimbursements, fees, funding, or salary from an organization that holds or has applied for patents relating to the content of the manuscript?
- Do you have any other financial competing interests?
- Do you have any non-financial competing interests in relation to this paper?

If you can answer no to all of the above, write 'I declare that I have no competing interests' below. If your reply is yes to any, please give details below.

I am co-founder of a company that creates FAIR-enabled software; however, I do not see this manuscript as putting me in CoI.

I agree to the open peer review policy of the journal. I understand that my name will be included on my report to the authors and, if the manuscript is accepted for publication, my named report including any attachments I upload will be posted on the website along with the authors' responses. I agree for my report to be made available under an Open Access Creative Commons CC-BY license (http://creativecommons.org/licenses/by/4.0/). I understand that any comments which I do not wish to be included in my named report can be included as confidential comments to the editors, which will not be published.

Choose an item.

To further support our reviewers, we have joined with Publons, where you can gain additional credit to further highlight your hard work (see: https://publons.com/journal/530/gigascience). On publication of this paper, your review will be automatically added to Publons, you can then choose whether or not to claim your Publons credit. I understand this statement.

Yes Choose an item.
