# Supplementary material for: Making experimental data tables in the life sciences more FAIR: a pragmatic approach
Source: Gigascience. 2020 Dec 15;9(12):giaa144. doi: 10.1093/gigascience/giaa144 (PMC7736789; doi:10.1093/gigascience/giaa144)
Supplement: giaa144_Reviewer_2_Report_Original_Submission — Martin Krzywinski, M.Sc. -- 8/6/2020 Reviewed [file giaa144_reviewer_2_report_original_submission.pdf]

Reviewer Report

Title: Towards FAIRification of experimental data tables in life sciences: a pragmatic approach

Version: Original Submission **Date:** 8/6/2020

Reviewer name: Martin Krzywinski, M.Sc.

Reviewer Comments to Author:

The authors provide a short summary of ways to make data publication compliant with FAIR principles (findable, accessible, interoperable, reusable). These principles are universally desirable and intuitive -- they describe requirements for sound research and communication of findings.
The paper describes ways of fixing the problem of spreadsheets without necessarily taking the spreadsheets away from the researchers. I would not agree that they are a "tool par excellence". They are convenient -- available and with a low bar for entry. Ironically, it is this very convenience that makes them unreliable for storage and analysis.
The paper hinges on 5 points, listed in the "Summary of proposed approach". My interpretation of these points is the following
1. Make data available through a service that is tailored to the needs of the researcher and their expertise in data wrangling.
2. Create a database schema, normalize the data and map it onto fixed vocabulary or ontology.
3. Train researchers to adopt FAIR principles a la carte and where it is both practical and useful for them to do so. Thus, as opposed to transitioning to a fully "FAIRized" system, stagger the adoption and training process and use the researcher's own data for training. Essentially, use a bit of social engineering to get people to do what you feel is good for them.
4. Have a way to assess whether implementing new data handling methods is adding benefit.
5. Every aspect of data collection, handling, analysis and publication should be designed with FAIR principles in mind.
These 5 points are true but, in my mind, they are so true that they are essentially widely accepted as necessary. For example, a fixed vocabulary and normalized database schema for data storage and lookup are common solutions to the problem of consistency and querying. I don't think that points like this need to be emphasized anymore.
The workflow shown in Figure 2 is flexible and sophisticated but illustrates steps that are required in any research venture that is sound (e.g. data backup, metadata, visualization).

Level of Interest

Please indicate how interesting you found the manuscript: Choose an item.

Quality of Written English

Please indicate the quality of language in the manuscript: Choose an item.

Declaration of Competing Interests

Please complete a declaration of competing interests, considering the following questions:

- Have you in the past five years received reimbursements, fees, funding, or salary from an organisation that may in any way gain or lose financially from the publication of this manuscript, either now or in the future?
- Do you hold any stocks or shares in an organisation that may in any way gain or lose financially from the publication of this manuscript, either now or in the future?
- Do you hold or are you currently applying for any patents relating to the content of the manuscript?
- Have you received reimbursements, fees, funding, or salary from an organization that holds or has applied for patents relating to the content of the manuscript?
- Do you have any other financial competing interests?
- Do you have any non-financial competing interests in relation to this paper?

If you can answer no to all of the above, write 'I declare that I have no competing interests' below. If your reply is yes to any, please give details below.

I declare that I have no competing interests

I agree to the open peer review policy of the journal. I understand that my name will be included on my report to the authors and, if the manuscript is accepted for publication, my named report including any attachments I upload will be posted on the website along with the authors' responses. I agree for my report to be made available under an Open Access Creative Commons CC-BY license (http://creativecommons.org/licenses/by/4.0/). I understand that any comments which I do not wish to be included in my named report can be included as confidential comments to the editors, which will not be published.

Choose an item.

To further support our reviewers, we have joined with Publons, where you can gain additional credit to further highlight your hard work (see: https://publons.com/journal/530/gigascience). On publication of this paper, your review will be automatically added to Publons, you can then choose whether or not to claim your Publons credit. I understand this statement.

Yes Choose an item.
