# Supplementary material for: Making experimental data tables in the life sciences more FAIR: a pragmatic approach
Source: Gigascience. 2020 Dec 15;9(12):giaa144. doi: 10.1093/gigascience/giaa144 (PMC7736789; doi:10.1093/gigascience/giaa144)
Supplement: giaa144_Reviewer_3_Report_Original_Submission — Pete McQuilton, PhD -- 8/14/2020 Reviewed [file giaa144_reviewer_3_report_original_submission.pdf]

Reviewer Report

Title: Towards FAIRification of experimental data tables in life sciences: a pragmatic approach

Version: Original Submission **Date:** 8/14/2020

Reviewer name: Pete McQuilton, PhD

Reviewer Comments to Author:

This well written paper aims to describe how researchers can prepare their data to be more FAIR, taking the specific example of plant data. The paper lays out a set of pragmatic guidelines to move the community closer to FAIRer data and good data management in general and concludes with an assessment of their test data via a number of recent FAIR data assessment frameworks.
There are many good pieces of advice in the paper and all reasoning is well explained. However, the focus on spreadsheet data, while understood, given spreadsheets are, in the words of the paper, 'the tool par excellence', does limit the scope somewhat, as some of the guidelines could be expanded to other types of data. However, given this focus, the paper would benefit from having more examples, explanations and recommendations of specific controlled vocabularies or reporting guidelines to follow (e.g. Why was ODAM chosen? Which CVs were chosen, are the CVs FAIR?)
I have two further specific points:
1. The authors choose (on line 90) the Frictionless Data JSON specifications as their interoperability standard of choice. This choice should be explained as many other interoperability standards are also available. This explanation could also touch on the use of metadata to place the dataset into context, a key factor to make the data FAIR, which is mentioned in the Frictionless Data specification but not in the main text.
2. One issue that hasn't been covered in the Publication of data section (line 98-115) is that of data licensing. This is an important issue in FAIR and should, in my opinion, be mentioned in this section.
For clarity, I would suggest the following additions to the text:
1. Line 90 - state the open, interoperability standard, rather than just the reference (so readers don't have to go to the reference list to find the name of the standard).
2. In general, the standards mentioned in the figures should also be mentioned in the text, as the figures seem slightly disconnected at present. For example, the use of ODAM in figure 1. ODAM isn't mentioned in the main text but is integral to the work.
There are a few typographical errors:
1. line 39 'e.g. *the* European commission'
2. line 59-60 'Thus, a tool *enabling the automatic combination of* data sets'
3. line 75 'because *they are* essentially using'
4. line 122 '2. *Researchers have* the best control'
The paper may also benefit from the addition of a reference on line 103 - 'Journal editors and reviewers are increasingly recommending that all data, complete and non-synthetic, generated during the course of the study be made available' - it would be good to provide a reference as evidence of this (I don't doubt the veracity, I just think it should be accompanied by a reference).

I'd like to conclude by congratulating the authors. I particularly enjoyed and agree with the statement that starts on line 94 - 'So, in our approach data FAIRification is closely related to data management, avoiding a retroactive process that would require more time, costs and computer skills'. This paper has many merits, not least as an example of how FAIR can and should be put into practice.

Level of Interest

Please indicate how interesting you found the manuscript: Choose an item.

Quality of Written English

Please indicate the quality of language in the manuscript: Choose an item.

Declaration of Competing Interests

Please complete a declaration of competing interests, considering the following questions:

- Have you in the past five years received reimbursements, fees, funding, or salary from an organisation that may in any way gain or lose financially from the publication of this manuscript, either now or in the future?
- Do you hold any stocks or shares in an organisation that may in any way gain or lose financially from the publication of this manuscript, either now or in the future?
- Do you hold or are you currently applying for any patents relating to the content of the manuscript?
- Have you received reimbursements, fees, funding, or salary from an organization that holds or has applied for patents relating to the content of the manuscript?
- Do you have any other financial competing interests?
- Do you have any non-financial competing interests in relation to this paper?

If you can answer no to all of the above, write 'I declare that I have no competing interests' below. If your reply is yes to any, please give details below.

I declare that I have no competing interests.

I agree to the open peer review policy of the journal. I understand that my name will be included on my report to the authors and, if the manuscript is accepted for publication, my named report including any attachments I upload will be posted on the website along with the authors' responses. I agree for my report to be made available under an Open Access Creative Commons CC-BY license (http://creativecommons.org/licenses/by/4.0/). I understand that any comments which I do not wish to be included in my named report can be included as confidential comments to the editors, which will not be published.

Choose an item.

To further support our reviewers, we have joined with Publons, where you can gain additional credit to further highlight your hard work (see: https://publons.com/journal/530/gigascience). On publication of this paper, your review will be automatically added to Publons, you can then choose whether or not to claim your Publons credit. I understand this statement.

Yes Choose an item.
